# Nitrous oxide for the treatment of depression: a systematic review and meta-analysis

**DOI:** 10.1016/j.ebiom.2025.106023

**Published:** 2025-11-30

**Authors:** Kiranpreet Gill, Angharad N. de Cates, Chantelle Wiseman, Susannah E. Murphy, Ella Williams, Catherine J. Harmer, Isabel Morales-Muñoz, Steven Marwaha

**Affiliations:** a Institute for Mental Health, University of Birmingham, Birmingham, UK; b Department of Psychiatry, University of Oxford, Oxford, UK; c Severe Mood Disorders Clinic, Birmingham and Solihull Mental Health NHS Trust, Birmingham, UK; d Oxford Health Foundation Trust, Warneford Hospital, Oxford, UK

**Keywords:** Nitrous oxide, N-methyl-d-aspartate receptor, Depression, Major depressive disorder, Treatment-resistant depression, Antidepressant

## Abstract

**Background:**

Depression remains a global public health challenge, prompting interest in translational targets which allow for more effective and rapidly acting interventions. Nitrous oxide (N2O), an N-methyl-d-aspartate receptor antagonist, has demonstrated potential as a rapid-acting antidepressant. This study synthesised existing data on the efficacy and safety of N2O in depressive disorders.

**Methods:**

We systematically reviewed clinical trials, exploratory studies, and protocol papers evaluating N2O for the treatment of depression, including major depressive disorder (MDD), treatment-resistant depression (TRD), and bipolar depression, following PRISMA guidelines. Meta-analysis was completed where possible. Primary outcomes were change in depressive symptoms and adverse events (AEs). Pooled mean differences (MD) and relative risk ratios were calculated using random- or fixed-effects models. Evidence mapping described trial characteristics across completed and ongoing studies.

**Findings:**

Seven clinical trials involving 247 participants with depressive disorders, and four protocol papers were reviewed. N2O was administered via inhalation at 25% or 50%, as single or repeated sessions, with comparators including air, oxygen, or midazolam. Pooled results from three trials administering 50% N2O in a single session showed significant reductions in depressive symptoms at 2 h (pooled MD −2.74, 95% Confidence Interval (CI): −4.72 to −0.76; p = 0.007) and 24 h (MD −3.32, 95% CI: −5.09 to −1.55; p < 0.0001), but not at 1 week post-inhalation (MD −1.52; 95% CI: −4.07 to 1.03; p = 0.24). AEs were mild and transient, with 25% N2O generally being better tolerated. Evidence mapping showed that most trials are early-phase and focused on short-term outcomes in adults with MDD and TRD.

**Interpretation:**

N2O demonstrates rapid, reproducible antidepressant effects in early-phase trials. Its future clinical value depends on whether these effects can be sustained over time through optimised dosing and extended/repeated use. Improved trial design, outcome standardisation, and population diversity is required to clarify its full potential for the treatment of depression.

**Funding:**

The funder had no role in study design, data collection, analysis, interpretation, or writing.

**Research in context**

**Evidence before this study**

Depression, encompassing major depressive disorder (MDD), treatment-resistant depression (TRD), and bipolar depression, is among the leading global causes of disability. First-line antidepressant medications often show limited efficacy in a substantial proportion of patients and are associated with a delayed onset of action. There is increasing interest in treatments that act more rapidly and target different neurobiological mechanisms implicated in depression. Nitrous oxide (N2O), an N-methyl-d-aspartate receptor antagonist, has demonstrated rapid antidepressant effects in early-phase trials. We searched PubMed, Embase, PsycINFO, Google Scholar, and ClinicalTrials.gov up to June 2025 for studies on N2O for depressive disorders. Previous reviews focused on short-term efficacy in small, early-phase trials, without examining ongoing studies or synthesising broader research trends such as trial design, populations studied, or outcome consistency.

**Added value of this study**

This review combines systematic review, meta-analysis, and evidence mapping to assess the therapeutic potential and research landscape of N2O for depression. Alongside pooled analyses confirming symptom reductions at 2- and 24-h post-inhalation, we applied structured evidence mapping to evaluate study design, dosing strategies, participant characteristics, and outcome measures across both completed and ongoing trials. This approach highlighted areas of concentrated activity, such as single-session trials, mostly in adults with MDD and TRD, as well as important gaps including repeated dosing, long-term outcomes, adolescent and bipolar populations, and lack of methodological standardisation.

**Implications of all the available evidence**

N2O shows promise as a rapid-acting intervention to alleviate depressive symptoms, particularly in MDD and TRD. However, further research is needed to address key gaps, improve standardisation, and determine long-term efficacy and safety. Evidence mapping offers a practical framework to identify research priorities and guide the development of scalable, clinically meaningful interventions.

## Introduction

Depression is a leading cause of disability worldwide, affecting over 300 million people with a lifetime prevalence of 16.2%.^1^, ^2^, ^3^, ^4^, ^5^ It involves complex dysregulation across biological, environmental and psychosocial systems, including neural networks.^6^ While monoamine abnormalities have been the traditional focus,^7^ disruptions in broader neuroregulatory systems including the hypothalamic–pituitary–adrenal axis, as well as functional changes in brain regions like the prefrontal cortex, also contribute to the pathophysiology of depression.^8^ Depression is also associated with poorer overall health outcomes compared to other chronic conditions such as arthritis, asthma or diabetes, including greater functional impairment and reduced quality of life.^9^ It imposes a substantial economic burden, with estimated costs of poor mental health exceeding €600 billion annually in Europe.^10^ First-line depression treatments, including selective serotonin reuptake inhibitors (SSRIs) and serotonin-noradrenaline reuptake inhibitors (SNRIs),^11^ are ineffective for 30–50% of patients,^12^^,^^13^ and typically take several weeks to exert clinical effects.^14^ These limitations have led to a growing interest in faster-acting, mechanistically distinct treatments, particularly for individuals with treatment-resistant depression (TRD),^6^ where standard treatments have repeatedly failed.^12^^,^^13^

**Fig. 1:**
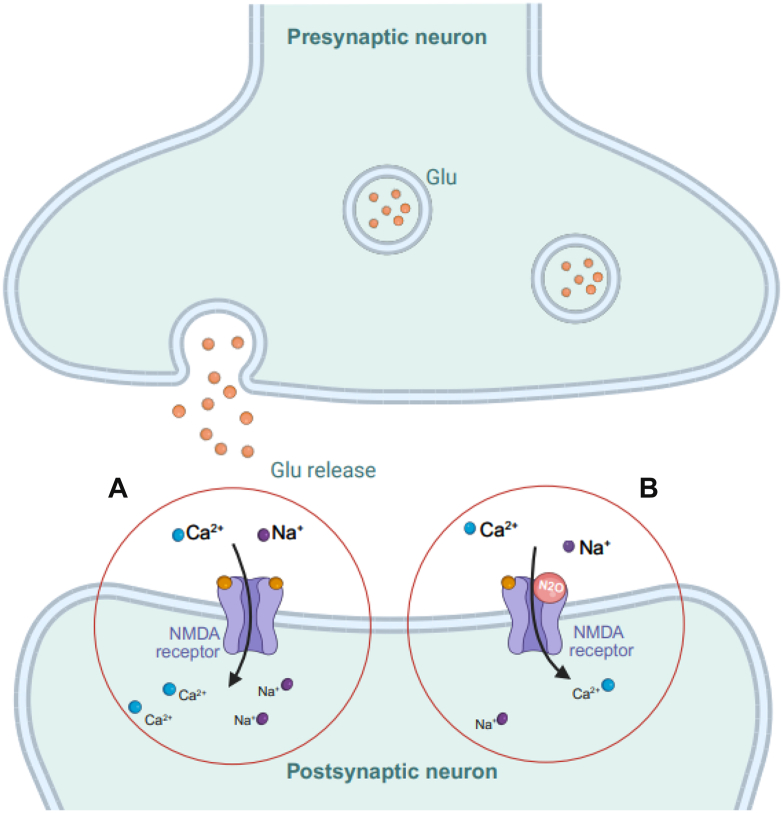
*Mechanism of action of nitrous oxide (N2O) in modulating NMDA receptor activity*. In the normal state (A), glutamate (*glu*) binds to NMDA receptors on the postsynaptic neuron, causing calcium (Ca^2+^) and sodium (Na^+^) ion influx, triggering excitatory signalling. In B) *N2O* partially blocks NMDA receptors, inhibiting glutamate binding and preventing Na^+^ and Ca^2+^ influx, thereby reducing excitatory signalling. This modulation of ions affects the excitatory and inhibitory balance in the central nervous system and is implicated in depression. Figure created with BioRender. Gill, K. (2025) https://BioRender.com/a87q343.

Over recent decades, treatment development has increasingly focused on targeting the glutamatergic system.^6^ For example, ketamine, a glutamate-modulating agent, has demonstrated rapid antidepressant effects, highlighting the potential of mechanisms beyond traditional monoaminergic pathways managing TRD.^15^^,^^16^ Its efficacy has catalysed interest in fast-acting treatments, prompting investigation into alternative N-methyl-d-aspartate (NMDA) receptor antagonists,^6^ such as nitrous oxide (N2O).^17^ Like ketamine, N2O modulates the glutamatergic system through NMDA receptor antagonism (see Fig. 1 for N2O's mechanism of action),^17^ and has traditionally been used as an anaesthetic and analgesic.^18^ N2O has similarly shown rapid-onset antidepressant effects,^17^ and in medical settings, its most commonly reported adverse effects include nausea, dizziness and headache, which are typically transient and dose-dependent.^17^^,^^19^ Its established mechanisms include NMDA receptor blockade,^20^, ^21^, ^22^ which restores disrupted glutamate signalling implicated in depression,^23^^,^^24^ and increases regional cerebral blood flow (CBF),^25^ potentially improving brain oxygenation and nutrient delivery.^26^

N2O may also reduce hyperconnectivity within the default mode network (DMN)/medial frontoparietal network, a neural network linked to self-referential thought and rumination in depression.^27^^,^^28^ Neuroimaging studies have observed reduced resting state functional connectivity between the subgenual anterior cingulate cortex and praecuneus, which are regions associated with negative self-referential processing and emotional dysregulation, in individuals with MDD who respond to N2O.^29^ Preclinical studies suggest this may reflect N2O's ability to restore activity in prefrontal circuits via NMDA-independent pathways, which subsequently leads to appropriate top-down suppression of the DMN.^30^

In addition, N2O modulates opioid and dopamine systems,^19^^,^^20^^,^^31^ both of which are central to affective regulation and reward sensitivity.^20^^,^^32^ Specifically, activation of central opioid receptors has shown to contribute to its antidepressant effects, with opioid antagonists attenuating its efficacy in both human and animal models.^29^^,^^33^ This likely reflects the opioid system's role in regulating emotional reactivity, hedonic tone and stress responsivity, which are commonly disrupted in depression.^34^^,^^35^ Dysregulation within these systems is implicated in core depressive symptoms,^34^^,^^36^ such as anhedonia,^37^ reduced motivation,^38^ and affective blunting,^39^ suggesting that N2O's broader neurochemical actions may supplement its glutamatergic mechanisms to alleviate depressive symptomatology.^20^^,^^32^

Previous systematic reviews and meta-analyses have examined the antidepressant effects of N2O, synthesising early-phase trials with small sample sizes and heterogeneous outcome measures.^40^, ^41^, ^42^ They focused on broader glutamatergic agents,^41^ or short-term outcomes.^40^^,^^42^ However, none have incorporated protocol papers, ongoing studies, and evidence mapping of trial characteristics across the research pipeline. This is needed to provide a comprehensive overview of current research activity, identify evidence gaps, and inform future study design, and clinical development of N2O as a treatment for depression.

### Aims and approach

This systematic review and meta-analysis aim to evaluate the efficacy, safety, and clinical relevance of N2O for depression, including major depressive disorder (MDD), TRD and bipolar depression (BD). Alongside completed clinical trials, it includes protocol papers and registered active interventional studies from ClinicalTrials.gov to capture ongoing research trends. Where data permitted, a meta-analysis was conducted to assess changes in depressive symptoms, and adverse events (AEs) relating to short-term tolerability. We also applied an evidence mapping approach to summarise study characteristics and identify gaps in the research pipeline.

## Methods

This review was conducted in accordance with the Preferred Reporting Items for Systematic Reviews and Meta-Analyses (PRISMA) guidelines,^43^ with eligibility criteria informed by the PICOS (Population, Intervention, Comparator, Outcome, and Study design) framework.^44^ The preregistered protocol is available via the Open Science Framework (osf.io/p4dw7).^45^

### Ethics

No ethical approval was sought for the conduct of this systematic review and meta-analysis, because no primary data collection involving human or animal subjects took place, and the data and analyses presented are based on previously published peer-reviewed research.

### Eligibility and data sources

We included studies that met the following criteria:

a) Evaluated the use of N2O for depression including MDD, TRD and BD;
b) Clinical trials (randomised or non-randomised), including early-phase or proof-of-concept studies;
c) Study protocols; and
d) Reported outcomes related to feasibility, efficacy, or safety.

Studies were excluded if:

a) They did not focus on depression;
b) They did not include or plan to assess outcomes related to N2O treatment; or
c) They were published in non-English languages.

We systematically searched PubMed, PsycINFO, and Embase, from database inception to June 2025. Google Scholar was also included to identify grey literature and additional studies not indexed in major databases, and the first 10 pages were screened to ensure the most relevant results were captured. We also manually screened reference lists of included articles and relevant reviews to identify additional eligible studies. Full search details are provided in Supplement 1. To capture ongoing research, we conducted a search of ClinicalTrials.gov (www.clinicaltrials.gov) through June 2025 using the term “nitrous oxide.” We included registered interventional trials of N2O for depression that were listed as not yet recruiting, recruiting, active but not recruiting, enrolling by invitation, or of unknown recruitment status. Trials were excluded if they were non-interventional or did not focus on depression.

### Screening and selection process

**Fig. 2:**
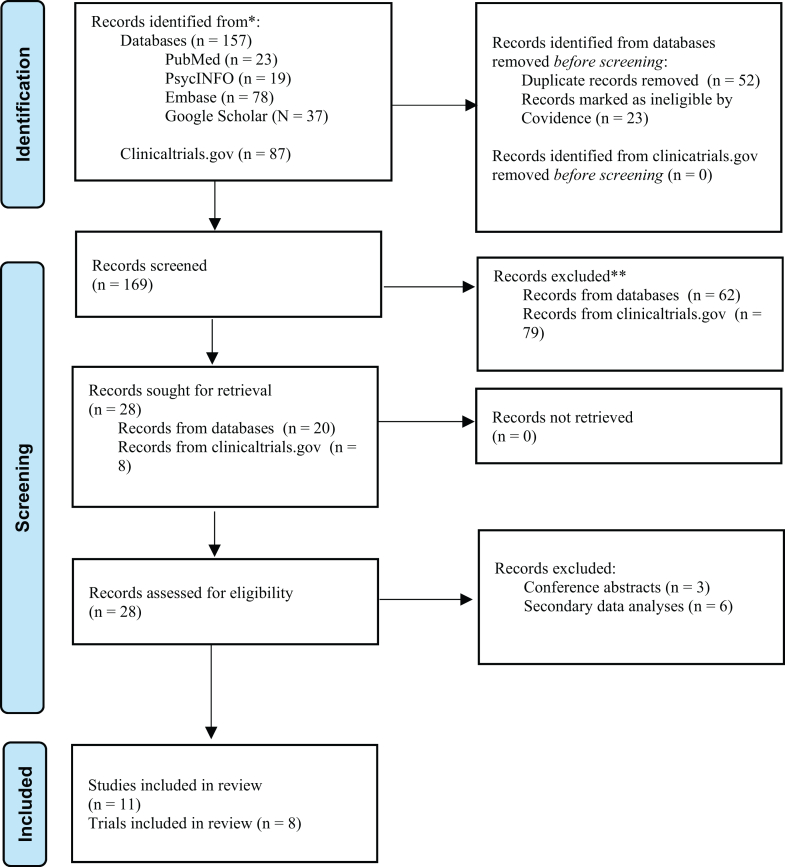
*PRISMA flow diagram*. Published studies (including completed clinical trials and exploratory research) and registered interventional trials (sourced from ClinicalTrials.gov) evaluating N2O for depressive disorders were identified, screened, and assessed for eligibility through database and registry searches conducted up to June 2025. Published studies refer to peer-reviewed clinical or exploratory research, and protocol papers. Registered trials include interventional studies listed on ClinicalTrials.gov. Articles automatically marked as ineligible by Covidence during database screening were manually reviewed by two independent screeners to confirm exclusion.

All articles were exported to Covidence (www.covidence.org) for screening. Two independent researchers (K.G. and A.N. de C.) screened titles and abstracts against predefined criteria, achieving 100% agreement without discrepancies. The screening and selection process is outlined in Fig. 2.

### Data extraction

Data were systematically collected using a standardised extraction form by K.G. and A.N. de C, capturing study design, N2O concentration, placebo type, intervention details, follow-up, participant characteristics, outcome measures, AEs, and key findings.

### Data synthesis

Studies were critically reviewed to assess N2O's efficacy and safety for depression. Findings were grouped by dosing regimens (single vs. repeated), concentrations (25% vs. 50%), and population (e.g. MDD). Data suitable for quantitative synthesis were analysed via meta-analysis methods, with the main outcome measures being pooled effect sizes (mean differences [MDs] with 95% confidence intervals [CIs]). Data that could not be included in the meta-analysis due to heterogeneity in study design, sample characteristics, dosing, and outcome measures, were examined through narrative synthesis. AEs data were grouped by N2O dose to evaluate tolerability.

Emerging and ongoing evidence was further evaluated using an evidence mapping approach, in accordance with PRISMA guidelines.^43^ This method systematically summarises the characteristics and scope of available studies and is used in areas where the evidence base is heterogeneous or still developing. Following the approach used by Vallarino et al. (2015),^46^ we reviewed key characteristics of both completed and active trials such as study design, phase, population, dosing, and outcomes measures, to evaluate the scope of existing research and identify gaps in the literature.

### Risk of bias assessment

Risk of bias was assessed using the Cochrane Risk of Bias 2 (RoB 2) tool,^47^ covering five domains rated as “low risk,” “some concerns,” or “high risk”. Studies were classified as high risk overall if any domain was rated high or if multiple domains raised concerns.

### Data analysis

Statistical analyses were performed using RevMan Web 5 (Cochrane Collaboration), with significance set at p < 0.050. All studies included were prospective in design. For continuous outcomes in controlled studies, pooled MDs and 95% CIs were calculated using the inverse variance method and Cohen's d. Only pre-crossover data were used in crossover trials to minimise carryover effects. For studies not reporting standard deviations (SDs) for change scores, SDs were imputed using a correlation coefficient of 0.7 between baseline and follow-up scores, following Cochrane guidance for meta-analysis of continuous outcomes.^48^ Where available, contrast-level data (MDs and standard error [SE]) were entered.

Dichotomous outcomes, including AEs, were analysed using relative risk (RR) via the Mantel–Haenszel method or inverse variance method under a random or fixed effects model, depending on heterogeneity. Heterogeneity was assessed using the I^2^ statistic, which was interpreted as low (<25%), moderate (25–50%), or high (>50%). For high heterogeneity, a Restricted Maximum-Likelihood based random-effects model with Hartung-Knapp-Sidik-Jonkman CIs was applied; otherwise, a fixed-effect model was used.

In the meta-analysis focussing on side effects, frequently reported AEs were pooled to assess N2O's tolerability. For open-label trials, AE data from N2O arms were extracted, assuming comparable baseline risk. Mean changes in depression scores were extracted or calculated to generate an aggregate graph visualising symptom reduction compared to baseline across studies. To visually assess potential publication bias at the 24-h timepoint, standardised mean differences (SMDs) and their SEs were calculated using pooled group SDs, or estimated from reported CIs, where necessary. A funnel plot was then generated using Stata version 19.5 (StataCorp).

### Role of the funding source

The funder of the study had no role in study design, data collection, data analysis, data interpretation, or writing of the report.

## Results

The search identified 157 articles through database searches, including one additional study retrieved via handsearching (Fig. 2). After removing 52 duplicates and 23 ineligible articles, 62 articles were excluded based on title and abstract screening (reasons provided in Supplement 2, Table S1), leaving 20 for full-text review. Of these, 11 met eligibility criteria, comprising of seven completed clinical trials,^29^^,^^49^, ^50^, ^51^, ^52^, ^53^, ^54^ and four published protocol papers (Table 1).^55^, ^56^, ^57^, ^58^ Results are presented according to study characteristics, efficacy outcomes (including pooled analyses), AEs, risk of bias, emerging trends from protocol papers and registered trials, and finally, evidence mapping to synthesise findings across literature.

**Table 1:** Summary of studies reviewed.

Study	Condition	Inclusion age	N	Comparison type	Study type	N2O Dose	N2O delivery method	Total sessions, duration and frequency	Relevant outcomes assessed	Data collection time points	Key results
Desmidt et al. (2023)^29^	Major depressive episode and resistant to at least 1 antidepressant, healthy controls	20–50	30	Equimolar mixture of oxygen and N2O	Open label trial	50%	EMONO bottles (4.4 m^3^ of 50% N2O/50% O2) with pressure regulator and 15 L integrated flow metre, delivered via high-concentration facial masks connected to a monitor (flow 9–12 L/min)	A single 60-min session per participant	MADRS, CGI, SSI, YMRS, CADSS, BPRS, QIDS-SR, brain MRI and ultrasound scan	Baseline, 24 h and 7 days post-treatment	Nine (45%) depressed participants were responders (≥50% reduction in MADRS at day 7), with median MADRS reductions of −18 (6) at 24 h and −19 (7) at day 7 in responders, compared to −9 (11) and −5 (7.5) in non-responders.
Dimick et al. (2020)^55^	BD	20–60	40	Placebo controlled (N2O and saline injection vs. medical air and midazolam injection)	RCT	25%	10% N2O/O2 for 5 min followed by 5 min washout, then 25% N2O/O2 for 20 min, followed by medical air flush delivered via face mask connected to a non-rebreathing circuit with 2 L reservoir. Saline injections (0.5 ml with 10% N2O and 1.5 ml with 25% N2O) given concurrently.	Single 20-min session per participant (N2O and saline or placebo)	SCID-Axis interview, MADRS, HDRS-17, BDI, YMRS, BPRS, CADSS, VAS, PRISE	Baseline, 2-, 3- and 7-days post-treatment	a
Guimarães et al. (2021)^51^	MDD	18–65	23	Placebo controlled (N2O vs. oxygen)	RCT	50%	N2O inhaled via disposable nasal mask connected to a portable N2O/O2 sedation device (Mandala-Matrix MDM, Porter Instruments, USA); flow between 5 and 7 L/min.	Eight 60-min sessions per participant (N2O or placebo) over 4 weeks, with two sessions per week	HDRS-17, BDI-II, YMRS, C-SSRS	Baseline, pre-treatment and post-treatment	Significant symptom improvement in N2O group (HDRS-17: 22.58–5.92) vs. placebo (22.44–12.89, p < 0.005); 91.7% achieved treatment response, 75% remission.
Kim et al. (2023)^53^	BD-I or BD-II with current major depressive episode	20–60	25	Placebo controlled (N2O and saline vs. air and midazolam)	RCT	25%	10% N2O/O2 for 5 min followed by 5 min washout, then 25% N2O/O2 for 20 min, followed by medical air flush delivered via face mask connected to a non-rebreathing circuit with 2 L reservoir. Saline injections (0.5 ml with 10% N2O and 1.5 ml with 25% N2O) given concurrently.	A single 20-min session per participant (N2O with saline or placebo)	MADRS, BDI, CADSS, VAS-D, PRISE, CBF via arterial spin labelling MRI	Baseline and 24 h post-treatment	N2O and midazolam both reduced MADRS scores significantly (N2O: −16.2 ± 6.1; midazolam: −14.6 ± 7.1, p = 0.56); higher response rate in N2O group at 120 min (92%) vs. midazolam (38%, p = 0.02); significant correlation in N2O group between lower baseline CBF and greater symptom reduction.
Ladha et al. (2023)^56^	MDD	18–65	40	Placebo controlled (N2O and saline vs. oxygen and midazolam)	RCT	50%	50% N2O/O2 inhaled via N2O delivery system with concurrent IV saline (100 ml) for 1 h. Once N2O flow stops, 100% O2 delivered for a few minutes.	Four 60-min sessions per participant (N2O or placebo) over 4 weeks, with one session per week	MADRS, QIDS, TSES	Baseline, post-treatment and at 6 weeks	a
Myles et al. (2025)^54^	MDD	≥18	81	Placebo controlled (N2O vs. oxygen/air)	RCT	25%, 50%	N2O delivered via the Porter N2O Sedation System connected to a scavenging system: 25% N2O/O2 via nasal mask or 50% N2O/O2 via face mask. Gas concentrations monitored with calibrated analyser. N2O titrated over 10 min to target level.	Four 60-min sessions per participant (N2O or placebo) over 4 weeks, with one session per week	HDRS-21, CAT-DI, CAT-SS	Baseline, pre- and post-treatment and at 4 weeks	N2O reduced HDRS scores more than control over four weeks (−1.9 points, 95% CI −3.9 to 0.0, p = 0.051); remission at week 1 was higher in the N2O group (38%) vs. control (13%, p = 0.031). N2O also significantly improved CAT-DI (−7.7 points, 95% CI −14.1 to −1.4, p = 0.017) and CAT-SS scores (−8.3 points, 95% CI −14.4 to −2.1, p = 0.008), both favouring N2O.
Nagele et al. (2015)^49^	TRD	18–65	20	Placebo controlled (N2O vs. air)	RCT	50%	Up to 50% N2O/50% O2 via standard anaesthesia facemask connected to an anaesthesia machine (flow 4–8 L/min). N2O titrated over first 10 min until 50% achieved, with inhaled/exhaled concentrations measured via facemask connector.	Two 60-min sessions (N2O and placebo), spaced one week apart	HDRS-21, QIDS-SR	Baseline, 2- and 24-h post-treatment	N2O significantly reduced depressive symptoms compared to placebo at 2 h (mean HDRS-21 difference: −4.8 points, p = 0.002) and 24 h (−5.5 points, p < 0.001).
Nagele et al. (2021)^50^	TRD	18–75	24	Placebo controlled (N2O vs. air/oxygen)	RCT	25%, 50%	N2O/O2 administered via standard anaesthesia face mask connected to anaesthesia machine or FDA-approved Porter/Praxair MXR breathing circuit (flow 2–8 L/min). N2O titrated over first 5–10 min, with inhaled/exhaled concentrations measured via facemask connector.	Three 60-min sessions per participant (25% N2O, 50% N2O, and placebo)	HDRS-21, MADRS, QIDS, CADSS, BPRS-18	Baseline, 2- and 24-h post-treatment, and 1- and 2-weeks post-treatment	N2O significantly improved symptoms compared to placebo (p = 0.01); no significant difference between 25% and 50% N2O (p = 0.58). 50% N2O reduced HDRS-21 more at week 2 (−7.00 points, p = 0.001); adverse events decreased with lower doses (p < 0.001).
Palanca et al. (2018)^57^	BD	18–75	64	Placebo controlled (N2O vs. nitrogen/oxygen)	Prospective, sequential parallel RCT	50%	Up to 50% N2O/50% O2 via standard anaesthesia facemask connected to anaesthesia machine or FDA-approved Porter/Praxair MXR breathing circuit (flow 2–8 L/min). N2O titrated over first 10 min to target concentration.	Stage 1: Three 60-min sessions (25% patients receiving N2O, 75% patients receiving placebo: 75% of patients), per week. Stage 2: Participants from Stage 1 placebo group receive three 60-min sessions (N2O or placebo) per week. Participants from Stage 1 N2O group continue with N2O.	HDRS-17, MADRS, YMRS, POMS 2, QIDS-SR, BPRS, SSI, CADSS	Baseline, 24 h post-inhalation of sessions 1 and 2. 72 h post-inhalation of session 3.	a
Stewart et al. (2017)^58^	MDD	12–17	30	Placebo controlled (N2O vs. nitrogen/oxygen)	RCT	50%	50% N2O/50% O2 via standard anaesthesia facemask connected to scavenged anaesthesia machine (flow 6 L/min).	Single 60-min session per participant (N2O or placebo)	HDRS-21, CDRS-R, BD1, WASI-II, K-SADS, YSR, CGI	Baseline, 2- and 24-h post-treatment, and 1–12 weeks post-treatment	a
Yan et al. (2022)^52^	TRD	18–60	44	Placebo controlled (N2O vs. air/oxygen)	RCT	50%	50% N2O/50% O2 via standard anaesthesia facemask connected to anaesthesia machine (flow 4–6 L/min). N2O titrated over first 10 min to target concentration.	A single 60-min session per participant (N2O or placebo)	HDRS-17, QIDS, VAS-D, CADSS	Baseline, 2- and 24-h post-treatment, and 1- and 2-weeks post-treatment	N2O significantly improved symptoms over two weeks vs. placebo (HDRS-17: −3.10 points at 2 h, p = 0.017; −3.19 points at 24 h, p = 0.033); higher response rate in N2O group (75%) vs. placebo (40.9%, p < 0.001), no significant remission differences. Interaction effect of time × group (F = 11.80, p = 0.002) showed greater reduction in depressive symptoms over 24 h in the N2O group.

MDD: Major Depressive Disorder; BD: Bipolar Disorder; BD-I: Bipolar Disorder Type 1; BD-II: Bipolar Disorder Type 2; TRD: Treatment Resistant Depression; RCT: Randomised Controlled Trial; MADRS: Montgomery-Asberg Depression Rating Scale; CGI: Clinical Global Impression; SSI: Scale for Suicidal Ideation; YMRS: Young Mania Rating Scale; CADSS: Clinician Administered Dissociative Scale; BPRS-18: Brief Psychiatric Rating Scale (18-item); QIDS: Quick Inventory of Depressive Symptomology; SCID-Axis Interview: Structured Clinical Interview for Axis 1 DSM-IV Disorders; HDRS (17/21): Hamilton Depression Rating Scale; BDI: Beck Depression Inventory; YMRS: Young Mania Rating Scale; VAS: Visual Analogue Scale; PRISE: Patient Rated Inventory of Side Effects; BDI-II: Beck Depression Inventory-II; C-SSRS: Columbia Suicide Severity Rating Scale; MRI: Magnetic Resonance Imaging; CBF: Cerebral Spinal Fluid; TSES: Toronto Side Effects Scale; CAT-DI: Computerized Adaptive Test-Depression Inventory; CAT-SS: Computerized Adaptive Test–Suicide Scale; POMS 2: Profile of Mood States Brief, 2nd edition; CDRS-R: Children's Depression Rating Scale-Revised; WASI-II: Wechsler Abbreviated Scale of Intelligence; K-SADS: Kiddie Schedule for Affective Disorders and Schizophrenia; YSR: Youth Self Report.

a Key results are not provided as these are protocol papers. “∗” denotes not applicable.

The seven completed randomised controlled trials (RCTs),^29^^,^^49^, ^50^, ^51^, ^52^, ^53^, ^54^ including two crossover trials,^49^^,^^50^ conducted across the United States, Brazil, China and Australia, enrolled 247 participants with MDD, TRD, or BD.^29^^,^^49^, ^50^, ^51^, ^52^, ^53^, ^54^ All trials assessed the efficacy of 25% and/or 50% N2O compared to placebo (air, oxygen or midazolam), delivered via inhalation for 20–60 min, typically in early-phase designs (Phase I–II or proof-of-concept). Most employed single-session protocols, with a subset investigating repeated administration.

Across trials, N2O consistently demonstrated rapid reductions in depressive symptoms. In a crossover trial by Nagele et al. (2015), 20 adults with TRD received two 60-min sessions of 50% N2O or placebo (air), administered 1 week apart.^49^ Significant reductions in Hamilton Depression Rating Scale-21 (HDRS-21) scores were observed in the N2O group at both 2 h (MD −4.8 points; p = 0.002) and 24 h (MD −5.5 points; p < 0.001) post-inhalation, which can be compared with the non-significant changes in the placebo group (2 h: MD −2.3 points; p = 0.14; 24 h: MD −2.8 points; p = 0.07). Treatment response (defined as ≥50% reduction in HDRS-21 score), was achieved for 20% of participants in the N2O group vs. 5% in the placebo group. Remission (HDRS-21 ≤ 7) was reached by 15% of participants in the N2O group, while none in the placebo group achieved remission. Yan et al. (2022) reported significant between-group differences in Hamilton Depression Rating Scale-17 (HDRS-17) scores in 44 patients with TRD after a single session of 50% N2O (vs. air/oxygen placebo).^52^ Estimated between-group differences favoured N2O at 2 h (−3.10 points, p = 0.017) and 24 h (−3.19 points, p = 0.033), though effects were not significant at 1 week (−1.28, p = 0.463). Desmidt et al. (2023) conducted an open-label study with a single session of 50% N2O in 30 participants, including healthy controls and patients with MDD who had shown resistance to at least one antidepressant treatment.^29^ Among depressed participants, 45% achieved a response (≥50% reduction in Montgomery-Åsberg Depression Rating Scale [MADRS]) at day 7. These responders exhibited greater symptom reductions than non-responders at both 24 h (median MADRS score:16 vs. 24) and day 7 (median MADRS score: 9 vs. 26). In BD, Kim et al. (2022) administered a single 25% N2O dose or placebo (air and midazolam) in 25 patients.^53^ Compared to placebo, N2O reduced MADRS scores at 24 h post-inhalation (−16.2 ± 6.1 vs. −14.6 ± 7.1, p = 0.56). Response rates were higher in the N2O group at both 2 h (92% vs. 38%) and 24 h (58% vs. 54%), but this difference was only statistically significant at 2 h (p = 0.02 vs. p = 0.91, respectively). Lower baseline regional CBF was associated with greater clinical improvement, suggesting a possible biomarker for treatment response.

Repeated dosing regimens of 60-min offered additional evidence of sustained antidepressant efficacy. Guimaraes et al. (2021) treated 23 patients with MDD, with eight sessions of 50% N2O or placebo (oxygen) over four weeks (twice weekly).^51^ The N2O group demonstrated significantly greater treatment response (≥50% reduction in HAMD-17 [91.7% vs. 44.4%, p = 0.046]) and remission rates (HAMD-17 < 7 [75% vs. 11.1%, p = 0.008]), at the end of the final (eighth) treatment session. In a crossover trial, Nagele et al. (2021) compared three sessions of 25% or 50% N2O vs. placebo (air/oxygen), in 24 patients with TRD.^50^ The combined 25% and 50% N2O groups showed a significant overall reduction in HDRS-21 scores vs. placebo over two weeks (p = 0.01). At week 2, estimated differences in HDRS-21 scores in N2O were −5.19 points for 25% N2O (p = 0.02) and −7.00 points for 50% N2O (p = 0.001) in comparison to placebo. Comparisons between doses at week 2 showed a −3.15 point decrease per 25% increase in dose (p = 0.001), favouring 50% N2O. Fewer AEs were reported with the 25% concentration. Most recently, Myles et al. (2025) randomised 81 patients with MDD to four, weekly sessions of either 25%, 50% N2O or placebo (air/oxygen).^54^ Compared to placebo, N2O improved depressive symptoms over the treatment period (HDRS-21: estimated MD −1.9 points, 95% CI: −3.9 to 0.0, p = 0.051), with greater symptom reductions 60 min after the final session (estimated MD −2.5 points, 95% CI: −5.9 to −0.1, p = 0.041). Comparisons between doses showed lower mean depression scores with 50% compared to 25% N2O across repeated sessions (e.g. 10.2 vs. 12.8 at week 4). Remission rates at week 1 were significantly higher in the N2O group compared to placebo (38% vs. 13%, p = 0.031). To illustrate these findings, Figure S1 (Supplement 3) presents a time-course comparison of symptom trajectories following N2O administration, using mean HDRS or MADRS scores (where available) at standardised timepoints post-inhalation (2 h, 24 h, 48 h, and 1 week) across studies. While most trials showed peak effects within 24–48 h, antidepressant effects were more enhanced and durable following repeated administration, suggesting a potential cumulative benefit.^51^^,^^54^

**Fig. 3:**
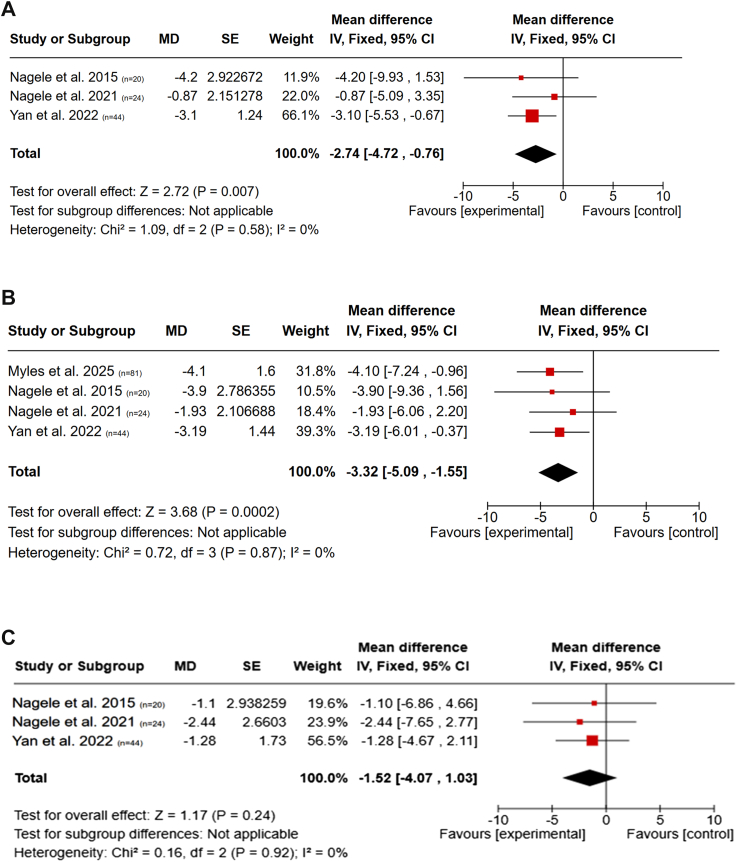
a–c. *Pooled effects of N2O on depression symptoms measured by HDRS at 2 h, 24 h, and 1-week post-inhalation*. Forest plots show pooled mean differences (MDs) in Hamilton Depression Rating Scale (HDRS) scores following N2O treatment compared to placebo at a) 2 h, b) 24 h, and c) 1-week post-inhalation. Analyses were conducted using the inverse variance method under a fixed-effect model. Statistically significant reductions in depressive symptoms were observed at 2 and 24 h, but not at 1 week. Heterogeneity was low across all timepoints (I^2^ = 0%). HDRS scores reflect HDRS-21 for Nagele et al. 2015,^49^ Nagele et al. 2021,^49^ and Myles et al. 2025,^54^ and HDRS-17 for Yan et al. 2022.^52^

In the meta-analysis (Fig. 3a–c), a pooled analysis of three studies reporting depression scores at 2 h post-inhalation with sufficient HDRS outcome data showed a significant antidepressant effect of N2O over placebo (MD −2.74; 95% CI: −4.72 to −0.76; p = 0.007; I^2^ = 0%), although differences were no longer significant at 1 week post-inhalation (MD −1.52; 95% CI: −4.07 to 1.03; p = 0.24).^49^^,^^50^^,^^52^ The antidepressant effect of N2O was also significant at 24 h post-inhalation (MD −3.32; 95% CI: −5.09 to −1.55; p < 0.001; I^2^ = 0%), based on four studies.^49^^,^^50^^,^^52^^,^^54^ Funnel plot analysis (Figure S2, Supplement 4) of four studies showed moderate asymmetry in the distribution of effect sizes at 24 h post-inhalation of 50% N2O,^49^^,^^50^^,^^52^^,^^54^ with one study reporting null or negative effects.^50^ This distribution may reflect the presence of publication bias.

AEs were generally mild, transient, and self-limiting, and no serious AEs (i.e. those requiring immediate medical intervention) were reported across all trials.^29^^,^^49^, ^50^, ^51^, ^52^, ^53^, ^54^ Common AEs included nausea, dizziness, headache, and transient dissociation, all resolving spontaneously. Nagele et al. (2021) observed fewer AEs with 25% N2O (11 events) compared to 50% (47 events),^49^ a pattern consistent with Myles et al. (2025).^54^ Similarly, Guimaraes et al. (2021) and Yan et al. (2022) found higher AEs in the N2O group compared with placebo group, although all were mild.^51^^,^^52^ There was no evidence that repeated exposure increased AE severity or frequency (see Table 2 for a full summary). Meta-analyses further quantified tolerability (see Figures S3a–5c, Supplement 5). Compared to placebo, 25% N2O was associated with a significantly higher risk of dizziness or light headedness (RR 2.79; 95% CI: 1.35–5.78; p = 0.006; I^2^ = 0%),^50^^,^^54^ and nausea and vomiting (RR 8.01; 95% CI: 1.61–39.77; p = 0.01; I^2^ = 0%).^50^^,^^53^^,^^54^ There were no significant differences in the incidence of headache (RR 1.14; 95% CI: 0.34–3.84; p = 0.84; I^2^ = 0%).^50^^,^^54^ Similarly, compared to placebo, 50% N2O significantly increased the risk of headache (RR 2.26; 95% CI: 1.08–4.72; p = 0.03; I^2^ = 38%),^49^^,^^50^^,^^52^^,^^54^ and dizziness or light headedness (RR 3.55; 95% CI: 1.92–6.56; p < 0.001; I^2^ = 42%),^49^^,^^50^^,^^54^ as well as nausea and vomiting (RR 12.69; 95% CI: 3.26–49.35; p < 0.001; I^2^ = 0%).^49^^,^^50^^,^^52^^,^^54^

**Table 2:** Summary of adverse events from studies reviewed.

	N2O dose	Short-term [i.e. during or immediately post-inhalation (N, %)]									
		Nausea/vomiting	Headache	Dizziness/light-headedness	Numbness/tingling	Feeling high	Anxiety	Claustrophobia	Regurgitation	Laughing	Long term
Desmidt et al. (2022)^29^	50%	7 (23%)	–	–	–	–	–	–	–	–	N/A
Guimaraes et al. (2021)^51^	50%	Yes	Yes	–	–	–	Yes	–	Yes	Yes	N/A
Kim et al. (2022)^53^	25%	1 (8%)	–	–	–	–	–	–	–	–	N/A
Myles et al. (2025)^54^	25% 50%	5 (25%) 8 (40%)	2 (20%) 7 (35%)	11 (55%) 16 (80%)	–	–	4 (20%) 7 (35%)	2 (10%) 5 (25%)	–	–	N/A
Nagele et al. (2015)^49^	50%	3 (15%)	2 (10%)	1 (5%)	2 (10%)	–	2 (10%)	1 (5%)	1 (5%)	–	N/A
Nagele et al. (2021)^50^	25% 50%	1 (5%) 7 (30%)	2 (10%) 4 (17%)	1 (5%) 5 (21.7%)	1 (5%) 3 (13%)	– 3 (13%)	– –	– –	– –	– 3 (13%)	25% N2O: Intestinal Gas 1 (5%); Common cold 2 (10%) 50% N2O: Common cold 3 (13%); Cramps 1 (4%); Sore throat 1 (4%)
Yan et al. (2022)^43^	50%	2 (9.1%)	1 (4.5%)	–	–	–	–	–	–	–	Headache 1 (4.5%); Tachycardia 2 (9.1%); Hypertension 1 (4.1%); Insomnia 2 (9.1)

Short-term adverse events refer to those occurring during or immediately after N2O inhalation. Long-term events reflect any adverse outcomes recorded days or weeks post-inhalation. Data are presented as number of participants (%), where available. “Yes” indicates the adverse event was reported but not quantified. “–” denotes not reported. Where studies tested both 25% and 50% N2O, adverse events are reported separately.

Direct comparisons of the risk of AEs between doses in two studies suggested improved tolerability with 25% N2O compared to 50% (see Figure S5a–c, Supplement 5).^50^^,^^54^ Although the difference in dizziness or light headedness did not reach statistical significance (RR 0.68; 95% CI: 0.42–1.08; p = 0.10; I^2^ = 0), and headache rates were similarly lower but non-significant (RR 0.39; 95% CI: 0.13–1.11; p = 0.08; I^2^ = 0%), the risk of nausea and vomiting was significantly lower with 25% compared to 50% N2O (RR 0.42; 95% CI: 0.18–0.97; p = 0.04; I^2^ = 35%).^50^^,^^54^ These findings suggest that while both doses increase the risk of certain AEs relative to placebo, 25% N2O may offer a more favourable tolerability profile.

Risk of bias assessment indicated two studies rated as low risk,^52^^,^^53^ three as having some concerns,^50^^,^^51^^,^^54^ and one as high risk,^49^ primarily due to potential carryover effects in the crossover design. Most domains, including randomisation, missing outcome data, and outcome measurement and reporting were consistently rated as low risk. Minor concerns were noted regarding maintenance of blinding, particularly in Myles et al. (2025) whereby approximately 74% of participants correctly guessed their treatment,^54^ and in some cases, the absence of intention-to-treat analyses. Full risk of bias assessment is provided in Supplement 6.

Four published protocol papers described planned RCTs examining N2O for depressive disorders (Table 1).^55^, ^56^, ^57^, ^58^ Stewart et al. (2017) planned to evaluate whether a single N2O session could improve mood in adolescents with MDD on SSRIs,^58^ though no updates have been reported to date. Palanca et al. (2018) planned a six-to eight-week trial of 50% N2O for treatment-resistant BD, but it was terminated due to low recruitment (n = 1).^57^ Dimick et al. (2020) investigated biomarkers of response to N2O or midazolam in BD, but this study was also terminated due to the COVID-19 pandemic,^55^ however, its findings were subsequently reported in the Kim et al. (2022) study.^53^ Finally, Ladha et al. (2024) designed a trial comparing weekly 50% N2O vs. midazolam over four weeks in patients with TRD, and the findings are currently under review.^56^

Emerging research trends were identified by reviewing active and registered clinical trials. Of the 87 N2O-related trials identified on clinicaltrials.gov, eight focused specifically on depressive disorders, including MDD, TRD, BD, and related suicidality. These were primarily Phase I-II RCTs testing 25–50% N2O or equivalents (EMONO, ENTONOX), typically delivered via inhalation in single or short-term protocols. Most trials assessed mood outcomes (e.g. HDRS, MADRS), with some incorporating biomarkers, cognitive tasks, or neurocognitive endpoints. Sample sizes ranged from 30 to 172, with several trials targeting high-risk or underrepresented populations such as older adults, individuals with suicidality or cognitive impairments. As of the latest search dated June 2025, four trials were actively recruiting, three were not yet recruiting, and one was enrolling by invitation (see Table S3, Supplement 7 for full details).

Finally, mapping of the evidence base across both completed and active studies revealed that research to date has largely focused on short-term, single-session protocols using 50% N2O in adults with MDD or TRD,^29^^,^^49^^,^^50^^,^^52^ with several ongoing trials continuing this approach. Repeated dosing has been explored in a small number of trials,^51^^,^^54^ and is under investigation in two trials. The largest of these is NCT05357040, which was registered to recruit 172 participants with MDD, comparing 25% and 50% N2O delivered weekly across four weeks. Although this trial remains listed as “recruiting” on Clinicaltrials.gov, it represents a duplicate registration of the Myles et al. (2025)^54^ study, which was conducted under a different site within the same multi-centre protocol, and was terminated early due to COVID-19 related disruptions. Adolescent populations remain underrepresented, with only one identified study protocol (Stewart et al. 2017),^58^ and no evidence of trial completion or active trials in this group. Meanwhile, research in BD is limited to a few small or prematurely terminated studies.^55^^,^^57^ Across both completed and ongoing studies, there is substantial variability in dosing protocols, session frequency, and outcome measures, limiting comparability and highlighting the need for greater standardisation (see Table 3 for full details).

**Table 3:** Evidence map of N2O trials for depression by population, dose, and dosing regimens.

Population/Condition	N2O dose	Dosing regimen	Completed trials	Ongoing trials	Evidence gaps
TRD	25%, 50%	Single session	Nagele et al. (2015),^49^ Yan et al. (2022)^52^	NCT05710887	Long-term efficacy not evaluated; no trials testing maintenance strategies
TRD	25%, 50%	Repeated	Nagele et al. (2021),^50^ Myles et al. (2025)^54^	NCT05007028	Few trials exploring repeated dosing beyond 4 weeks; limited biomarker tracking
MDD	50%	Repeated	Guimaraes et al. (2021),^51^ Myles et al. (2025)^54^	NCT05357040 (25% & 50%)	Dose comparison in large samples limited; relapse prevention unknown
MDD	50%	Single/Booster	–	NCT03736538 (4 sessions and 2 boosters)	Effect of booster sessions not yet published; tolerability over time unconfirmed
MDD with cognitive impairment	50% (EMONO)	Repeated (3 sessions)	–	NCT06382389	Underrepresented population; cognitive endpoints rarely studied
BD	25%, 50%	Single session	Kim et al. (2023)^53^	–	Few trials in BD; no repeated dosing; unclear if N2O works as monotherapy
Adolescents with MDD	50%	Single session	–	Stewart et al. (2017; no results)^58^	No completed adolescent trials; no efficacy or AE data
Suicidal ideation (any depression type)	50%	Single session	–	NCT06430489, NCT06636357	Emerging area; need data on acute vs. sustained effects
Healthy controls/mixed	50%	Single session	Desmidt et al. (2023)^29^	–	Open label only; used primarily for exploratory biomarkers
General depression	50% (ENTONOX)	Single session	–	NCT06557642	Novel cognitive outcomes (emotional/cognitive tasks); no clinical symptom follow-up

This table provides an overview of completed and ongoing clinical trials investigating N2O for depressive disorders. Trials are grouped to highlight where evidence exists, where studies are in progress, and where significant evidence gaps remain. Completed trials refer to published studies with available results, while ongoing trials correspond to active or registered studies on ClinicalTrials.gov. This evidence map is intended to guide interpretation of the current clinical landscape and inform future research priorities in N2O-based treatments for depression.

## Discussion

This systematic review and meta-analysis evaluated the efficacy, safety, and clinical relevance of N2O as a treatment for depression, including MDD, TRD and BD. Across seven studies involving 247 participants, most investigated a single-session of 25% or 50% N2O for 20–60 min.^29^^,^^49^, ^50^, ^51^, ^52^, ^53^, ^54^ Depressive symptoms were assessed at timepoints ranging from 2 h to 1-week post-inhalation. Pooled analysis of four studies reporting HDRS outcomes showed statistically significant reductions in symptoms compared to placebo (air/oxygen) at 2 h (MD −2.74; 95% CI: −4.72 to −0.76; p = 0.007),^49^^,^^50^^,^^52^ and at 24 h (MD −3.32; 95% CI: −5.09 to −1.55); p < 0.0001),^49^^,^^50^^,^^52^^,^^54^ effects were not sustained beyond 1 week (MD −1.52; 95% CI: −4.07 to 1.03; p = 0.24).^49^^,^^50^^,^^52^ N2O was generally well tolerated, though mild and transient AEs such as nausea, dizziness and headache were more common with higher doses (50% N2O).^29^^,^^49^, ^50^, ^51^, ^52^, ^53^, ^54^

The transient nature of symptom relief highlights the need for dosing regimens capable of sustaining clinical benefit. Repeated administration was associated with more durable reductions in depressive symptoms compared to single-session protocols.^51^^,^^54^ A time-course comparison across studies (Figure S1, Supplement 3) supports this, showing that single-session effects typically peak between 2- and 48-h, and diminish by 1-week, while repeated dosing produces more sustained responses. Evidence from two studies trialling both 25% and 50% N2O suggests a possible dose–response effect.^50^^,^^54^ Nagele et al. (2021) reported significantly greater reductions in depression scores with 50% N2O at week 2,^50^ while Myles et al. (2025)^54^ observed lower mean depression scores with 50% N2O across repeated sessions, without direct comparisons between doses. Although 25% N2O was associated with fewer AEs, and may be more tolerable for maintenance treatment, its clinical utility could be constrained by lower response and remission rates observed in some studies compared to 50%.^51^^,^^54^

Evidence mapping of completed and ongoing trials showed that research is generally concentrated around early-phase studies in adults with MDD or TRD, using single-dose protocols. In contrast, trials investigating repeated or maintenance dosing, longer-term outcomes, adolescent and bipolar populations, and consistent outcome frameworks remain limited.

Ketamine represents a relevant comparator for N2O, given their shared glutamatergic mechanism and ketamine's established use in severe depression.^6^^,^^17^ Meta-analyses of trials administering 0.5 mg/kg intravenous (IV) ketamine over 30–40 min show significant reductions in depressive symptoms at 24 h (SMD 0.68; 95% CI 0.46–0.90), which decline by 1 week (SMD 0.49).^6^ In comparison, N2O produced significant antidepressant effects at 24 h (MD −3.32, 95% CI: −5.09 to −1.55; p < 0.0001), but not at 1 week (MD −1.52; 95% CI: −4.07 to 1.03; p = 0.24) post-inhalation. Although both act as non-competitive NMDA receptor antagonists,^17^ ketamine produces rapid antidepressant effects via NMDA receptor antagonism on inhibitory interneurons, resulting in glutamate release, AMPA receptor activation and downstream synaptic plasticity.^17^^,^^59^^,^^60^ It is also associated with greater dissociation, reflecting stronger and more sustained NMDA receptor blockade.^61^ In contrast, N2O produces partial and weak voltage-dependent NMDA inhibition, and also modulates calcium-channel,^24^ and opioid systems,^29^ consistent with its more transient yet better-tolerated AEs. These pharmacological differences may contribute to the apparent shorter duration of antidepressant effects observed with N2O, compared to IV ketamine.

Clinical response rates also appear higher for ketamine (50–70%),^62^ than for a single dose of 50% N2O (20–45%) at 24 h.^29^^,^^49^ Both agents are associated with central nervous system-related AEs, with dissociation, psychotomimetic symptoms and cardiovascular changes more common with ketamine,^6^ whereas nausea, vomiting and headaches are frequently reported with N2O.^29^^,^^49^, ^50^, ^51^, ^52^, ^53^, ^54^ However, the relative incidence of nausea and vomiting remains uncertain due to the lack of direct comparative data between N2O and ketamine. Reported differences may reflect variation in dosing, delivery methods, or study populations rather than true pharmacological disparities. Further, while ketamine has been studied more extensively, the reliability of its evidence base is limited because systematic reviews have been rated as “critically low” quality on A Measurement Tool to Assess Systematic Reviews (AMSTAR-2), due to inadequate risk of bias assessments and poor reporting.^6^ These differences are summarised in Table S4 (Supplement 8). These findings should be interpreted with caution, particularly when contrasting ketamine with the smaller but emerging body of evidence on N2O. Moreover, ketamine trials have mainly recruited patients with TRD and multi-drug resistance,^6^ whereas N2O studies have included broader depressive populations (MDD, TRD, BD).^29^^,^^49^, ^50^, ^51^, ^52^, ^53^, ^54^ Comparisons therefore highlight N2O's potential within the landscape of rapid-acting antidepressants, but interpretation remains limited by methodological differences.^63^ These include the use of different effect size metrics (SMDs vs. MDs), heterogenous study populations, variable outcome measures, and differences in treatment delivery (IV vs. inhalation).

Previous reviews synthesised early findings from a small number of early-phase trials,^40^, ^41^, ^42^ focussing on broader glutamatergic agents,^41^ or early efficacy outcomes.^40^^,^^42^ Some conducted timepoint-specific analyses of depressive symptoms and/or selected AEs,^41^^,^^42^ but none incorporated active protocols or recent clinical studies. By integrating completed trials, protocol papers, and ongoing studies up to June 2025, this review provides a more comprehensive and current evaluation of N2O's therapeutic potential, while evidence mapping highlights both where research has concentrated, and where gaps remain.

Interpretation of findings is limited by several methodological factors across the evidence base. For example, only two studies reported a priori power calculation,^52^^,^^54^ the remaining were small, early-phase trials without formal sample size determination, reducing power and increasing uncertainty around effect estimates. A further limitation is variability in depression scales, outcome definitions, assessment timepoints, and control strategies across studies. The type of control condition is an important consideration, as inert gas comparators may risk unblinding and heighten expectancy effects, whereas active comparators like midazolam may help preserve blinding, and yield more conservative estimates of treatment effects.^64^ Such differences add further complexity to cross-study comparisons.

Another potential source of variability lies in treatment delivery systems, as N2O administration requires specialised equipment.^32^^,^^65^ Studies differed in delivery methods used and level of detail reported (Table 1), ranging from anaesthesia machines and FDA-approved breathing circuits to nasal-mask devices, non-rebreathing circuits, and fixed-dose cylinders. Such heterogeneity may affect dosing precision and the concentration inhaled, with potential implications for treatment outcomes. Most studies also focused on short-term outcomes following a single N2O session, leaving major gaps in knowledge regarding repeated dosing or longer-term efficacy. Finally, design differences, concomitant treatments, and heterogeneous populations further limit generalisability of current findings. Taken together, these factors highlight limitations of the existing evidence base, restricting certainty and generalisability of the conclusions that can be drawn.

This review itself also has limitations. The small number of studies and modest sample sizes limited the power of the meta-analysis and prevented detailed examination of sub-group differences, dose–response effects, or publication bias. A funnel plot was included to explore potential bias at the 24-h timepoint, but with only four studies, its interpretability is limited, and any observed asymmetry should be interpreted with caution. Our analyses were further constrained by the quality and reporting of included studies, meaning that certain outcomes such as vomiting distinct from nausea, could not be evaluated separately, though the distinction is important.

Future research should directly address these limitations identified to advance the clinical development of N2O as a treatment for depressive disorders. First, larger, methodologically rigorous RCTs are needed to confirm the durability of N2O's antidepressant effects beyond short-term improvements, and to evaluate longer-term safety. The Myles et al. (2025),^54^ study and its duplicate registration (NCT05357040) represented the largest planned RCT to date with a target sample of 172 participants, designed to investigate repeated dosing and direct comparisons of 25% vs. 50% N2O. Records indicate that 81 participants have been recruited to date, which remains the largest sample in an existing study. Despite providing important early insights, the premature termination of this multicentre study means that large-scale investigations, designed to have enough power to detect dose response effects, durability of efficacy, or other critical factors, are still lacking. This gap limits certainty regarding sustained efficacy, optimal dosing, and longer-term safety, all of which are important for clinical translation and shaping evidence-based practice. Second, dedicated trials should clarify whether N2O acts to augment existing antidepressants or is a standalone treatment.

Third, optimisation of dosing schedules, maintenance strategies, and dose–response relationships is critical to assess sustained clinical benefits. Two completed trials have evaluated maintenance strategies such as repeated booster sessions or extended dosing schedules,^51^^,^^54^ but the likelihood of attenuated treatment effects developing with repeated exposure remains unclear. Delivery methods may also influence dosing accuracy. For example, lower concentrations of N2O (25%) typically requires specialist mixing systems,^66^ whereas 50% is often delivered via fixed-dose cylinders,^67^ which may enhance dosing consistency and reduce leakage risk. Addressing these methodological concerns and clarifying whether N2O's antidepressant effects diminish or persist with ongoing use, will be essential to inform safe and effective maintenance protocols, and ensure treatment fidelity. Fourth, greater consistency in outcome measurement is needed to improve comparability across studies. Harmonisation of depression rating scales, and uniformity in assessment timepoints, and definitions of treatment response and remission would strengthen future meta-analyses and facilitate the development of clear, evidence-based clinical guidelines.

Fifth, assessment of blinding and its credibility are important in demonstrating the effects of N2O in depression because expectancy effects, as recognised in psychedelic research,^64^ may similarly bias N2O treatment outcomes. Of the seven completed studies, only three assessed blinding.^50^^,^^53^^,^^54^ Two found that most participants correctly guessed their treatment allocation (≥75%),^50^^,^^54^ while one reported accuracy close to chance levels.^53^ Together, this suggests that blinding was only partially successful and importantly, none of the studies assessed pre-treatment expectations. Routine evaluation of both pre-treatment expectations and post-treatment guesses of treatment allocation would strengthen internal validity, and reduce the risk of expectancy-related bias. Sixth, developing personalised treatment approaches should be prioritised to predict individual responses and refine patient selection. Although emerging trials have begun incorporating biomarker assessments, most remain exploratory, with limited application for stratifying patients or predicting outcomes. Standardised biomarker sets and longitudinal tracking of biomarker–symptom relationships may help identify reliable predictors of treatment response and clarify N2O's mechanisms of action in depression.

Beyond biological mechanisms, ongoing trials are evaluating N2O as a rapid-onset, in-clinic intervention in outpatient settings, and enrolling more diverse clinical populations, including those with suicidality, cognitive impairments, and comorbid anxiety. However, inclusion of high-risk and comorbid groups remains limited. Clarifying N2O's therapeutic potential in patients with both depression and anxiety, where evidence suggests possible dual efficacy,^19^^,^^68^ could enhance its clinical applicability.

While the findings of this review support the potential of N2O as a rapid-acting treatment for depression, adequately powered trials are essential to establish its long-term efficacy, safety, and acceptability. Recreational misuse is reported by 8.7% of 16 to 24-year-olds in the UK,^69^ often involving high cumulative doses of N2O at concentrations >89%,^70^^,^^71^ increasing the risk of vitamin B12 deficiency, cognitive dysfunction, and megaloblastic anaemia.^72^, ^73^, ^74^, ^75^ These effects stem from N2O-induced inactivation of vitamin B12, essential for cellular metabolism and DNA synthesis.^76^ High concentrations can also cause hypoxia (reduced oxygen supply to tissues),^77^ prompting guidelines recommending oxygen co-administration.^78^ Although typically reversible with supplementation and cessation,^70^ frequent misuse raises dependency concerns,^79^ necessitating structured and strict clinical protocols.

In conclusion, N2O shows promise as a rapid-acting intervention for depression, addressing an urgent need for faster-acting antidepressant treatments. Although early studies demonstrate consistent short-term improvements, uncertainties regarding long-term efficacy, optimal dosing, and its role as a standalone treatment remain. Future research must address methodological inconsistencies, clarify underlying mechanisms, and systematically evaluate N2O's feasibility across diverse, real-world populations. Rigorous and standardised clinical trials are essential to strengthen the evidence base for N2O and establish its role within the evolving landscape of antidepressant therapies.

## Contributors

K.G., S.M., and I.M.M. developed the scope and specification of the work. S.M. and I.M.M. provided K.G. with intellectual input throughout all stages of study development and conduct. K.G. led the writing of the manuscript, including database searches, article retrieval, analysis, and figure generation. K.G. and A.N.de C. conducted screening and risk-of-bias assessments, with A.N.de C. also reviewing and validating the analysis. A.N.de C., I.M.M., and S.M. provided critical revisions, while C.W., S.E.M., E.W., and C.J.H. contributed valuable feedback. All authors reviewed and approved the final manuscript.

## Data sharing statement

This study is based on published data from primary studies. Data derived for pooled analyses are available upon reasonable request from the corresponding author.

## Declaration of interests

K.G. is a Managing Editor for Trials (journal). S.M and A.N.de C receive support from the British Association of Psychopharmacology to attend their Summer Meeting and grant funding from the NIHR TRC Mental Health Mission. S.M also receives grant funding from NIHR HTA. A.N.de C has previously received fellowship support from The Wellcome Trust and The Guarantors of Brain. She also is a Section Editor/Editorial Board Member for the British Journal of Psychiatry and a member of the Royal College of Psychiatrists Psychopharmacology Committee. C.J.H has received consultancy fees from P1vital Ltd, J&J, Ieso, UCB, and Lundbeck, and holds grant income from J&J. S.E.M. has received consultancy fees from UCB and holds grant income from Zogenix, J&J and ADM. E.W. holds a contract with Compass Pathways.
