# Supplementary figures and images for: Nitrous oxide for the treatment of depression: a systematic review and meta-analysis
Source: eBioMedicine. 2025 Nov 30;122:106023. doi: 10.1016/j.ebiom.2025.106023 (PMC12790589; doi:10.1016/j.ebiom.2025.106023)

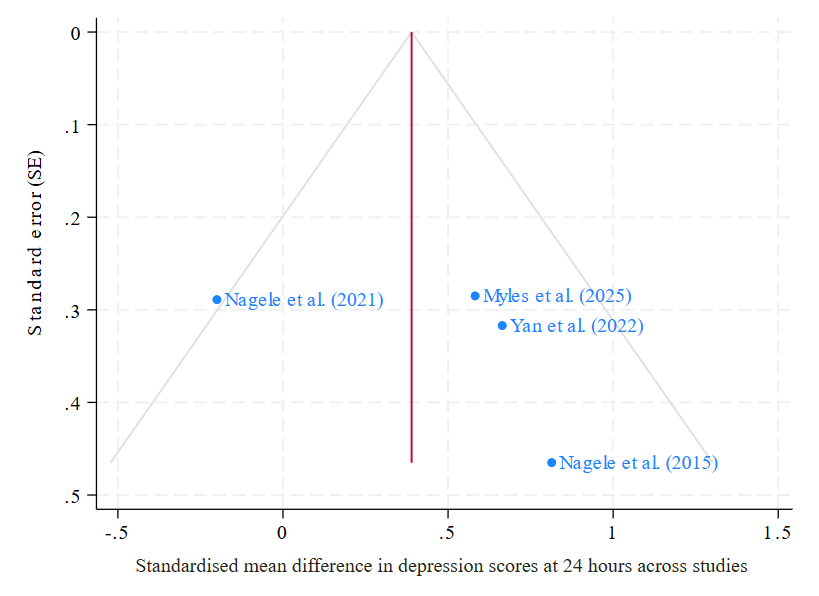

Supplement: Supplementary Figure S2 [file mmc2.docx]

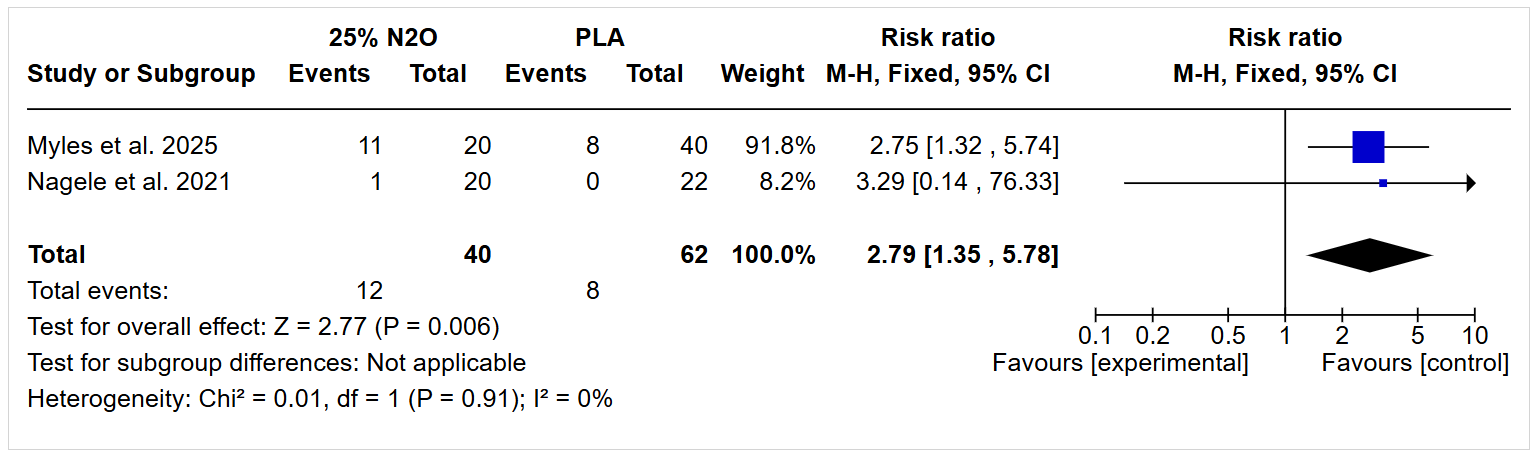


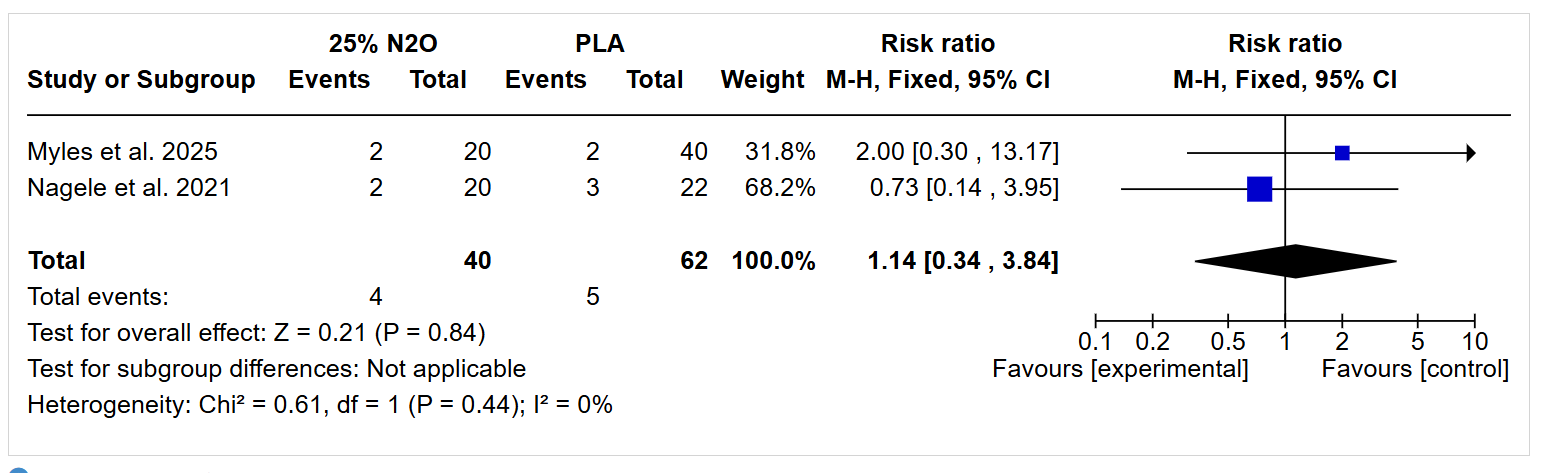


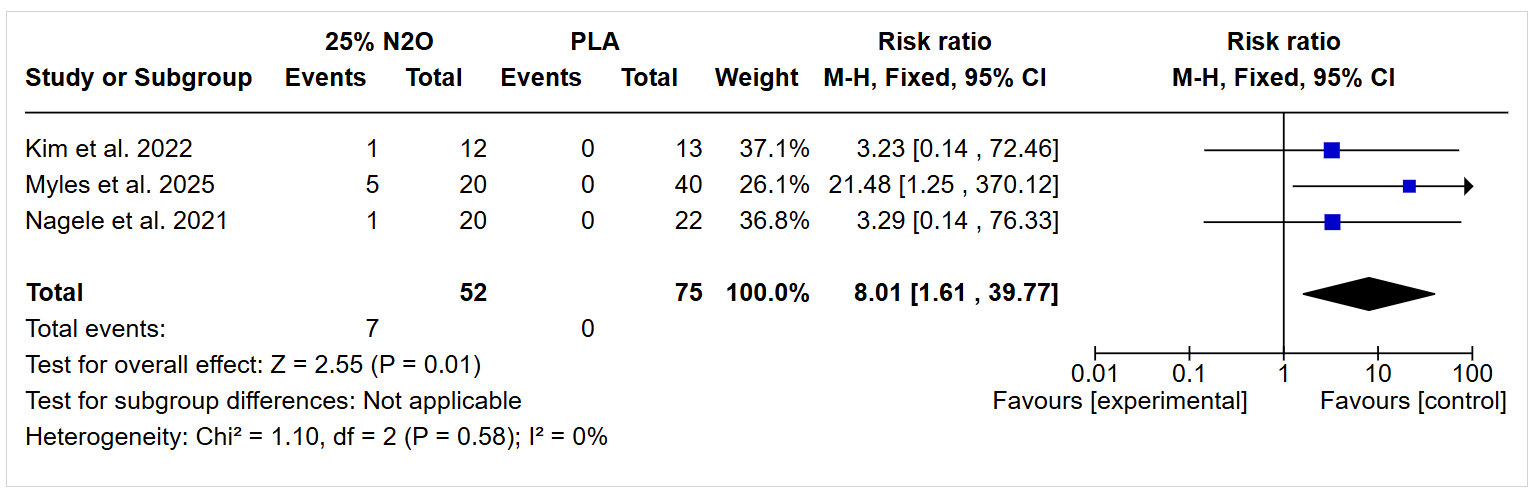


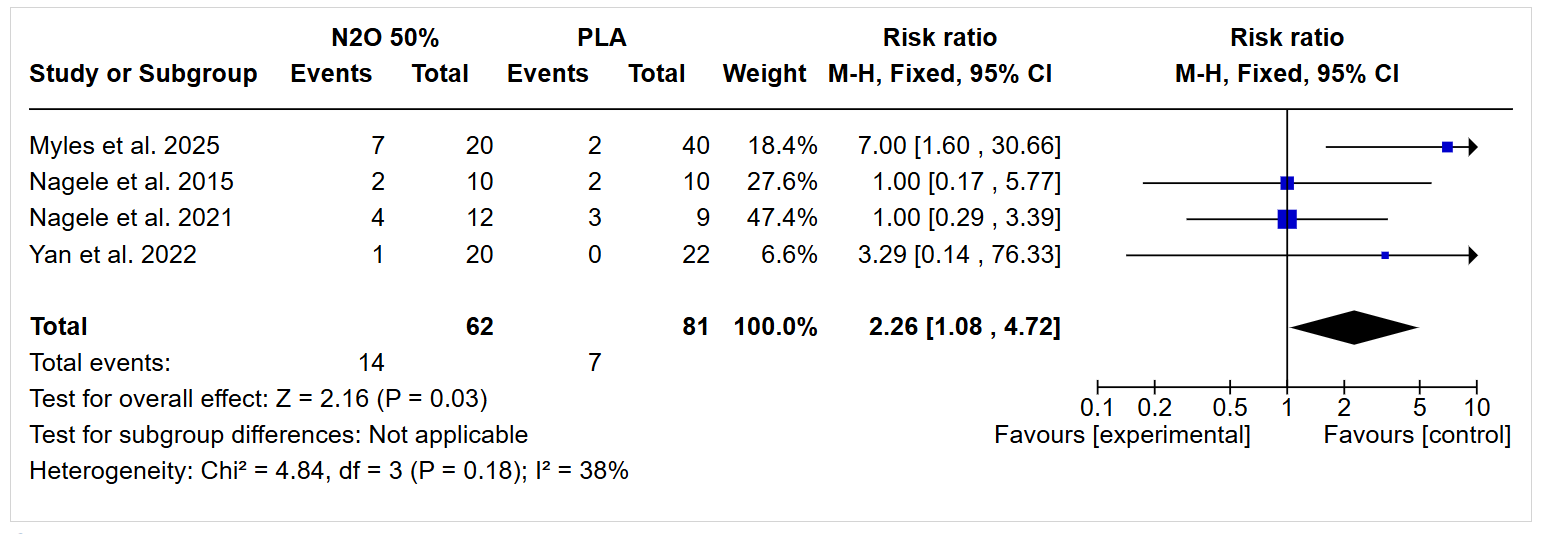


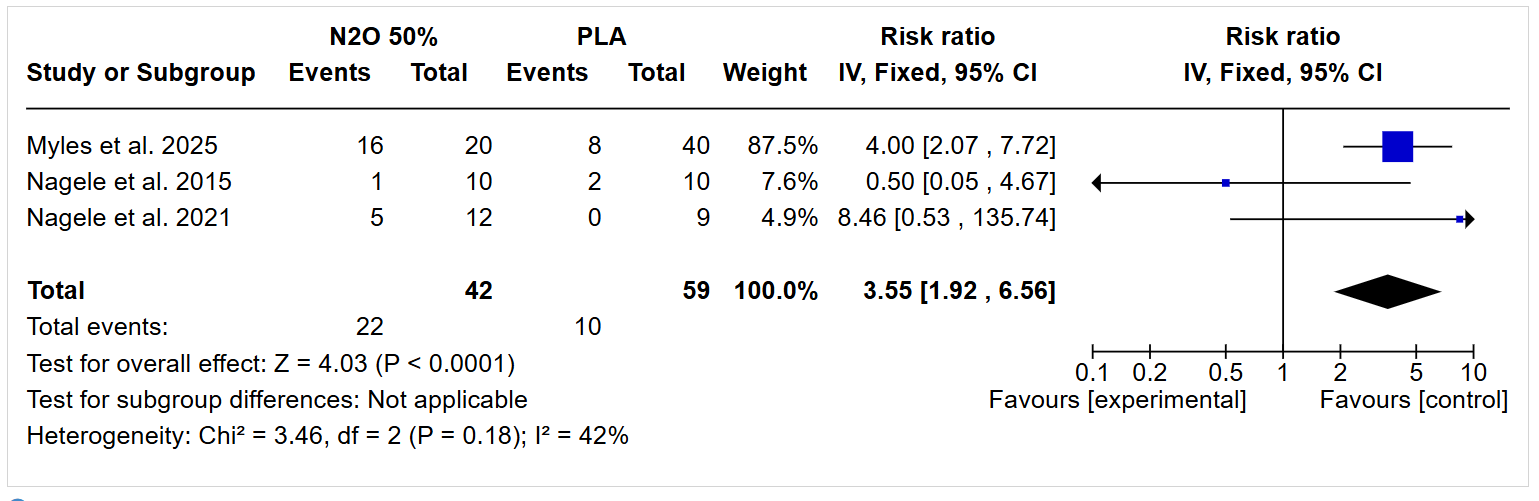


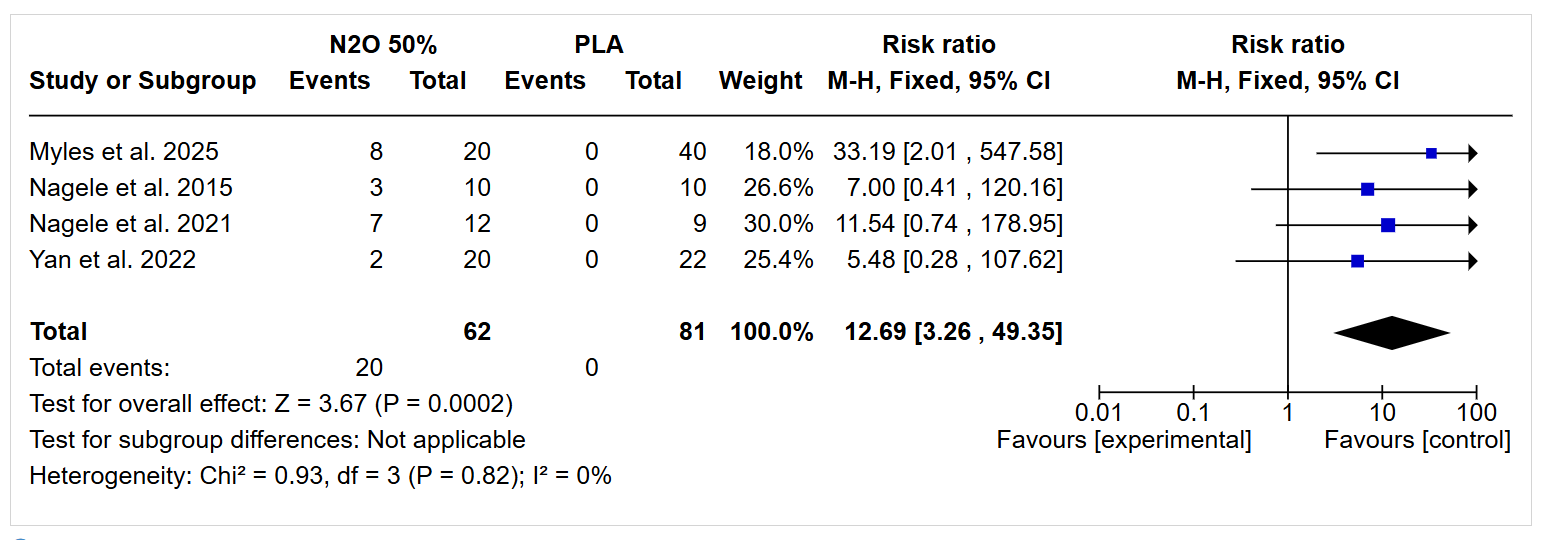


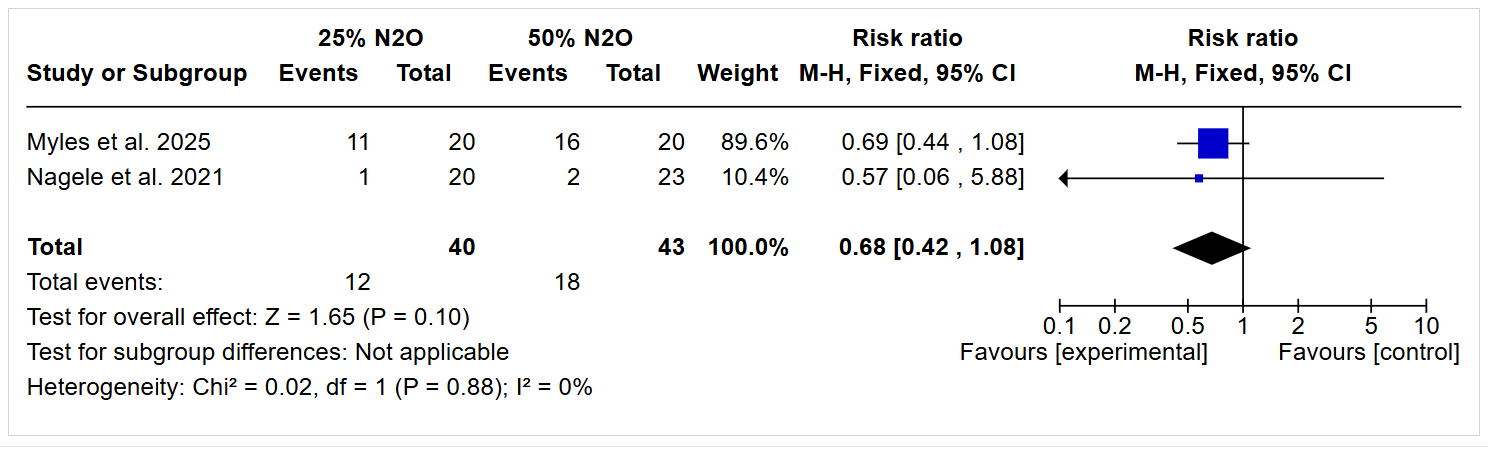


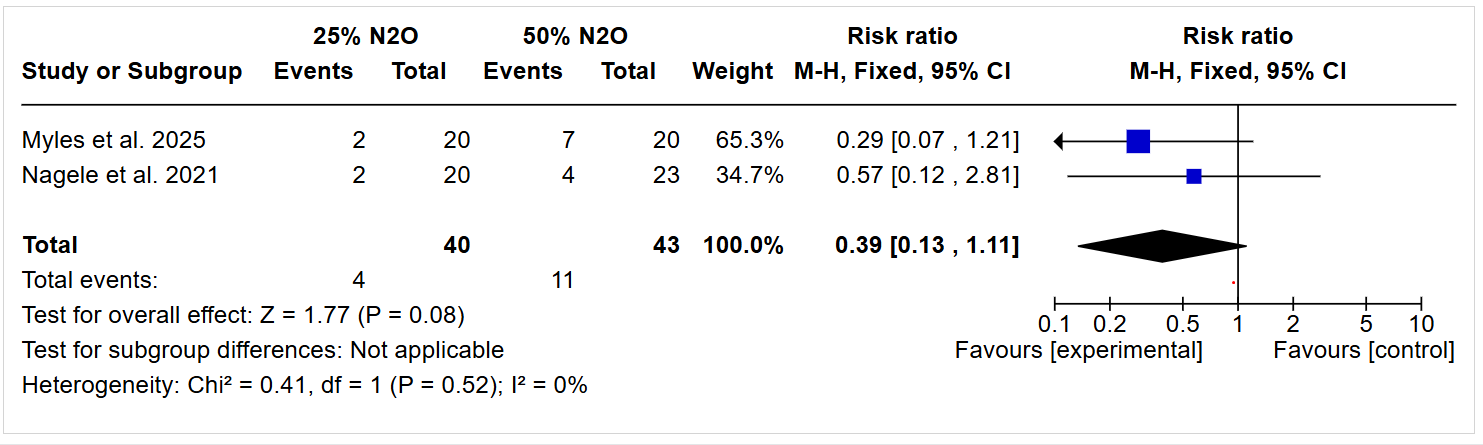


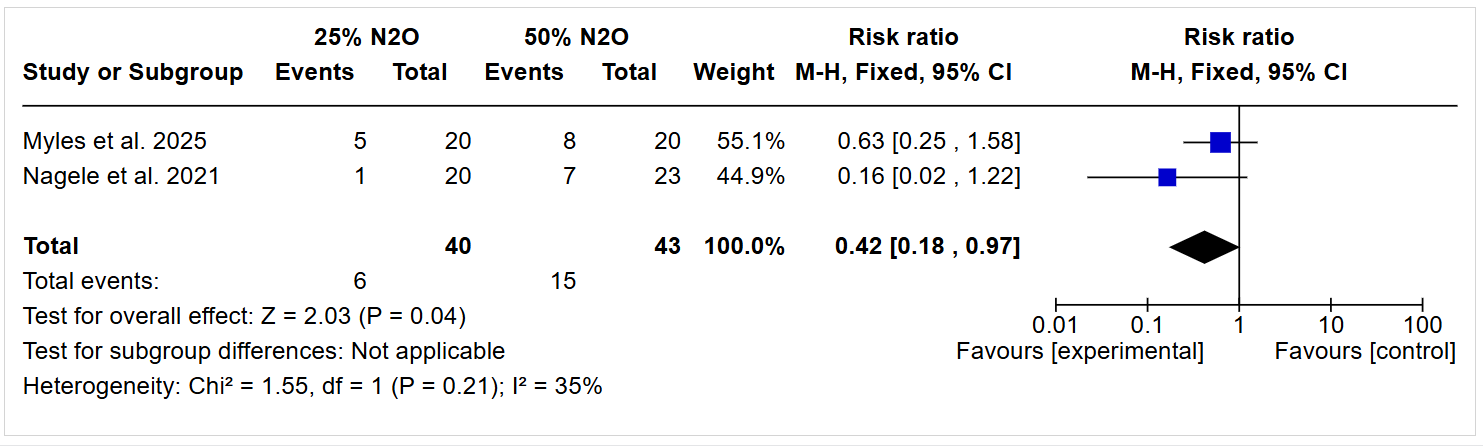

Supplement: Supplementary Figures 3a–5c [file mmc3.docx]
