# Supplementary material for: Nitrous oxide for the treatment of depression: a systematic review and meta-analysis
Source: eBioMedicine. 2025 Nov 30;122:106023. doi: 10.1016/j.ebiom.2025.106023 (PMC12790589; doi:10.1016/j.ebiom.2025.106023)
Supplement: Supplementary Material 6 [file mmc4.docx]

Guimarães et al. (2021),^52^ Yan et al. (2022),^53^ Kim et al. (2023),^54^ and Myles et al. (2025),^55^ were evaluated using the Cochrane Risk of Bias (RoB) 2 tool for randomised controlled trials [(RCTs) Figure S6a)],^48^ while Nagele et al. (2015),^50^ and Nagele et al. (2021),^51^ were assessed using the RoB 2 tool for crossover trials (Figure S6b).^48^ Overall, Yan et al. (2022),^53^ and Kim et al. (2023),^54^ were rated as low risk of bias, Guimarães et al. (2021),^52^ Nagele et al. (2021),^51^ and Myles et al (2025),^55^ as having some concerns, and Nagele et al. (2015),^50^ as high risk. Across all five studies, Domain 1 (randomisation), Domain 3 (missing outcome data), Domain 4 (measurement of outcomes), and Domain 5 (selection of reported results) were all classified as low risk. Each study employed appropriate randomisation methods, maintained high retention rates, used validated depression rating scales with blinded assessors, and reported predefined outcomes without evidence of selective reporting.

Blinding was implemented in all studies, however, the psychoactive effects of N2O make complete blinding challenging. While some participants may have recognised their treatment, no major deviations from protocol were reported, resulting in a low-risk classification across most studies. Nagele et al. (2021),^51^ Kim et al. (2023),^54^ and Myles et al. (2025),^55^ formally measured the accuracy of participants guessing their treatment allocation, while the other studies did not, though this did not impact overall risk ratings. In Nagele et al. (2021),^51^ and Kim et al. (2023),^54^ guessing rates were near chance levels and did not impact overall risk ratings. In contrast, Myles et al. (2025),^55^ reported a substantially higher correct guess rate (approximately 74%), resulting in a classification of some concerns in Domain 2, although outcome assessors remained blinded.

For Guimarães et al. (2021),^52^ the absence of an intention-to-treat (ITT) analysis led to an overall classification of some concerns in Domain 2, as two dropout participants were excluded from the final analysis. According to Cochrane RoB 2 guidelines,^49^ exclusions without ITT can introduce attrition bias, as excluding participants post-randomisation may affect the comparability of groups. While in this case group comparability remained intact, ITT is considered the preferred approach to maintain methodological robustness, justifying the classification as “some concerns”.

In Myles et al. (2025),^55^ missing outcome data were appropriately handled through multiple imputation, using chained equations, consistent with CONSORT and Cochrane guidelines to account for missing data.^81^ Dropout rates were low and similar across groups, with 5.1% in the 25% N2O group (2/39 participants), 7.4% in the 50% N2O group (3/40 participants), and 5.1% in the placebo group (2/39 participants), supporting a low-risk rating for Domain 3 (missing outcome data).

For the crossover trials, Nagele et al. (2015),^50^ was classified as high risk in Domain S due to a significant carryover effect (p = 0·02) and an insufficient washout period, which was not fully adjusted for in the primary analysis. This rating aligns with Cochrane guidelines,^35^ which indicate that when a carryover effect is detected and not adequately accounted for, the risk of bias should be considered high, as treatment effects from the first period may influence results in the second period. In contrast, Nagele et al. (2021),^51^ did not detect a significant carryover effect and implemented a sufficient washout period. However, as the washout period was not formally validated with statistical methods, Domain S was rated as "some concerns", in line with Cochrane guidance,^49^ which suggest that when the adequacy of a washout period is assumed rather than statistically tested, residual treatment effects cannot be ruled out with certainty.
