# Supplementary material for: Nitrous oxide for the treatment of depression: a systematic review and meta-analysis
Source: eBioMedicine. 2025 Nov 30;122:106023. doi: 10.1016/j.ebiom.2025.106023 (PMC12790589; doi:10.1016/j.ebiom.2025.106023)
Supplement: Supplementary Table S1 [file mmc5.docx]

| **Number of excluded Studies/Total** | **Cause** | **Reference** |
| --- | --- | --- |
| 21 | Ineligible because they are literature/narrative/systematic reviews | - Farber, N.B., 2019. NMDA Antagonists for Treatment-Resistant Depression. Handb Exp Pharmacol 250, 287–305. https://doi.org/10.1007/164_2018_165 - Henter, I.D., de Sousa, R.T., Zarate, C.A.J., 2018. Glutamatergic Modulators in Depression. Harvard Review of Psychiatry 26, 307. https://doi.org/10.1097/HRP.0000000000000183 - Ionescu, D.F., Papakostas, G.I., 2016. Current Trends in Identifying Rapidly Acting Treatments for Depression. Curr Behav Neurosci Rep 3, 185–191. https://doi.org/10.1007/s40473-016-0075-4 - Kalmoe, M.C., Janski, A.M., Zorumski, C.F., Nagele, P., Palanca, B.J., Conway, C.R., 2020. Ketamine and nitrous oxide: The evolution of NMDA receptor antagonists as antidepressant agents. J Neurol Sci 412, 116778. https://doi.org/10.1016/j.jns.2020.116778 - Lew, V., McKay, E., Maze, M., 2018. Past, present, and future of nitrous oxide. Br Med Bull 125, 103–119. https://doi.org/10.1093/bmb/ldx050 - Nagele, P., Zorumski, C.F., Conway, C., 2018. Exploring Nitrous Oxide as Treatment for Mood Disorders: Basic Concepts. J Clin Psychopharmacol 38, 144–148. https://doi.org/10.1097/JCP.0000000000000837 - Quach, D.F., de Leon, V.C., Conway, C.R., 2022. Nitrous Oxide: an emerging novel treatment for treatment-resistant depression. J Neurol Sci 434, 120092. https://doi.org/10.1016/j.jns.2021.120092 - Tadler, S.C., Mickey, B.J., 2018. Emerging evidence for antidepressant actions of anesthetic agents. Current Opinion in Anesthesiology 31, 439. https://doi.org/10.1097/ACO.0000000000000617 - Thase, M.E., 2017. New medications for treatment-resistant depression: a brief review of recent developments. CNS Spectrums 22, 39–48. https://doi.org/10.1017/S1092852917000876 - Zarate, C.A., Machado-Vieira, R., 2015. Potential Pathways Involved in the Rapid Antidepressant Effects of Nitrous Oxide. Biological Psychiatry, Depression and Immune Mechanisms 78, 2–4. https://doi.org/10.1016/j.biopsych.2015.04.007 - Zorumski, C.F., Nagele, P., Mennerick, S., Conway, C.R., 2015. Treatment-Resistant Major Depression: Rationale for NMDA Receptors as Targets and Nitrous Oxide as Therapy. Front Psychiatry 6, 172. <https://doi.org/10.3389/fpsyt.2015.00172> - Leal, B., Vila-Chã, D., Garcia, S., Pinto, I., Mateiro, R., Avelino, M., Martins, M. and Salgado, J., 2022. Nitrous Oxide in Treatment Resistant Major Depression: Should We Laugh About It?. *European Psychiatry*, *65*(S1), pp.S716-S716. - Gillman, M.A., 2019. Mini-review: a brief history of nitrous oxide (N2O) use in neuropsychiatry. *Current Drug Research Reviews Formerly: Current Drug Abuse Reviews*, *11*(1), pp.12-20. - van Amsterdam, J., Nabben, T. and van den Brink, W., 2015. Recreational nitrous oxide use: prevalence and risks. *Regulatory toxicology and pharmacology*, *73*(3), pp.790-796. - Liu, H., Kerzner, J., Demchenko, I., Wijeysundera, D.N., Kennedy, S.H., Ladha, K.S., Bhat, V., 2022. Nitrous oxide for the treatment of psychiatric disorders: A systematic review of the clinical trial landscape. Acta Psychiatr Scand 146, 126–138. https://doi.org/10.1111/acps.13432 - Sakurai, H., Yonezawa, K., Tani, H., Mimura, M., Bauer, M., Uchida, H., 2022. Novel Antidepressants in the Pipeline (Phase II and III): A Systematic Review of the US Clinical Trials Registry. Pharmacopsychiatry 55, 193–202. <https://doi.org/10.1055/a-1714-9097> - Shamabadi, A., Ahmadzade, A., Aqamolaei, A., Mortazavi, S.H., Hasanzadeh, A. and Akhondzadeh, S., 2022. Ketamine and Other Glutamate Receptor Modulating Agents for Treatment-Resistant Depression: A Systematic Review of Randomized Controlled Trials. *Iranian Journal of Psychiatry*, *17*(3), p.320. - Qiu, Y., Li, L., Duan, A., Wang, M., Xie, M., Chen, Z. and Wang, Z., 2023. The efficacy and tolerability of inhaled nitrous oxide in major depressive disorder: a systematic review and meta-analysis. *Psychopharmacology*, *240*(10), pp.2033-2043. - Breault, M.S., Orguc, S., Kwon, O., Kang, G.H., Tseng, B., Schreier, D.R. and Brown, E.N., 2025. Anesthetics as Treatments for Depression: Clinical Insights and Underlying Mechanisms. *Annual Review of Neuroscience*, *48*. - Mielko, Joana, Julia Pakulska, Amelia Oszczyk, Klaudia Lustyk, Karolina Pytka, and Kinga Sałaciak. "Beyond surgery: Repurposing anesthetics for treatment of central nervous system disorders." *Progress in Neuro-Psychopharmacology and Biological Psychiatry* (2025): 111386. - Philip, Annlin Bejoy, Janette Brohan, and Basavana Goudra. "The role of GABA receptors in anesthesia and sedation: an updated review." *CNS drugs* 39, no. 1 (2025): 39-54. |
| 3 | Ineligible because they are studies with healthy patients or studies without patients diagnosed with major depressive disorder or treatment-resistant depression | - Kamboj, S.K., Zhao, H., Troebinger, L., Piazza, G., Cawley, E., Hennessy, V., Iskandar, G., Das, R.K., 2021. Rewarding Subjective Effects of the NMDAR Antagonist Nitrous Oxide (Laughing Gas) Are Moderated by Impulsivity and Depressive Symptoms in Healthy Volunteers. Int J Neuropsychopharmacol 24, 551–561. <https://doi.org/10.1093/ijnp/pyab009> - Piazza, Giulia G., Georges Iskandar, Vanessa Hennessy, Hannah Zhao, Katie Walsh, Jeffrey McDonnell, Devin B. Terhune, Ravi K. Das, and Sunjeev K. Kamboj. "Pharmacological modelling of dissociation and psychosis: an evaluation of the Clinician Administered Dissociative States Scale and Psychotomimetic States Inventory during nitrous oxide (‘laughing gas’)-induced anomalous states." *Psychopharmacology* 239, no. 7 (2022): 2317-2329. - Hong, H.Y., Karadaghy, O., Kallogjeri, D., Brown, F.T., Yee, B., Piccirillo, J.F. and Nagele, P., 2018. Effect of nitrous oxide as a treatment for subjective, idiopathic, nonpulsatile bothersome tinnitus: a randomized clinical trial. *JAMA Otolaryngology–Head & Neck Surgery*, *144*(9), pp.781-787. |
| 3 | Ineligible because they are animal studies | - Chamaa, F., Bahmad, H.F., Makkawi, A.-K., Chalhoub, R.M., Al-Chaer, E.D., Bikhazi, G.B., Nahas, Z., Abou-Kheir, W., 2018. Nitrous Oxide Induces Prominent Cell Proliferation in Adult Rat Hippocampal Dentate Gyrus. Front Cell Neurosci 12, 135. https://doi.org/10.3389/fncel.2018.00135 - Izumi, Y., Hsu, F.-F., Conway, C.R., Nagele, P., Mennerick, S.J., Zorumski, C.F., 2022. Nitrous Oxide, a Rapid Antidepressant, Has Ketamine-like Effects on Excitatory Transmission in the Adult Hippocampus. Biol Psychiatry 92, 964–972. <https://doi.org/10.1016/j.biopsych.2022.06.016> - Alitalo, O., Kohtala, S., Rosenholm, M., Saarreharju, R., González-Hernández, G., Sarparanta, M., Rozov, S. and Rantamäki, T., 2024. Nitrous oxide induces hypothermia and TrkB activation: Maintenance of body temperature abolishes antidepressant-like effects in mice. *Neuropharmacology*, *261*, p.110172. |
| 6 | Ineligible because they are case report studies | - Sayyab A.M., Sondhi N., Deras B., Hirsch A.R., 2017. Chronic nitrous oxide use and seasonal affective disorder. JNP 29, e1–e2. <https://doi.org/10.1176/appi.neuropsych.16110299> - Desmidt, T., Gissot, V., Dujardin, P.A., Andersson, F., Barantin, L., Brizard, B., Arlicot, N., Réméniéras, J.P., Espitalier, F., El-Hage, W. and Camus, V., 2021. A case of sustained antidepressant effects and large changes in the brain with a single brief exposure to nitrous oxide. *The American Journal of Geriatric Psychiatry*, *29*(12), pp.1298-1300. - Wang, S., Cheng, S., Feng, M., Guo, P., Qian, M., Shen, X., Chen, R. and Wang, G., 2020. Sevoflurane augmentation in treatment-resistant depression: a clinical case study. *Therapeutic Advances in Psychopharmacology*, *10*, p.2045125320957126. - Aleberteau, M., Gallet, Q., Madieta, L., Desmidt, T., Riquin, E., Poisson, G.B., Gohier, B. and Kazour, F., 2025. Nitrous oxide reduced suicidal ideation in bipolar postpartum depression–A case report. *L'Encéphale*. - Yoon, Sojung, Dong Yu Kim, and Min Kyung Chu. "Nitrous oxide abuse unmasking anti-phospholipid syndrome in a 24-year-old male with cerebral venous thrombosis and pulmonary thromboembolism: a case report." *BMC neurology* 25, no. 1 (2025): 1-6. - Provaznikova, Barbora, Sebastian Olbrich, Erich Seifritz, Maximilian Dominik Haas, and Golo Kronenberg. "Sustained remission of treatment-resistant depression following two inhalations of nitrous oxide: A case report." *Psychiatry Research Case Reports* (2025): 100261. |
| 4 | Ineligible because they are conference abstracts | - Conway, C., 2019. 123. Inhaled Nitrous Oxide as a Rapid Acting Antidepressant. Biological Psychiatry, 74th Annual Scientific Convention and Meeting 85, S51. https://doi.org/10.1016/j.biopsych.2019.03.137 - Conway, C., Nagele, P., 2017. 417. Recent Trial Data from Nitrous Oxide Effects in Treatment-Resistant Depression. Biological Psychiatry 81, S170. <https://doi.org/10.1016/j.biopsych.2017.02.901> - Desmidt, T., 2023. FC33: Hilarious Gas for treatment resistant depression in older adults: is it really serious?. *International Psychogeriatrics*, *35*(S1), pp.96-97. - Conway, C.R. and Nagele, P., 2015, May. Nitrous Oxide for TRD: Preliminary Findings. In *BIOLOGICAL PSYCHIATRY* (Vol. 77, No. 9, pp. 321S-322S). 360 PARK AVE SOUTH, NEW YORK, NY 10010-1710 USA: ELSEVIER SCIENCE INC. |
| 1 | Ineligible because they are secondary data analyses providing no additional information for the studies included in the review | - de Leon, V.C., Kumar, A., Nagele, P., Palanca, B.J., Gott, B., Janski, A., Zorumski, C.F. and Conway, C.R., 2023. Nitrous oxide reduced suicidal ideation in treatment-resistant major depression in exploratory analysis. *The Journal of clinical psychiatry*, *84*(5), p.48436. |
| 16 | Ineligible because they were correspondence/opinion pieces/letters to the editor/editorials that did not present original data. | - Fluegge, K., 2020. Nitrous Oxide (N2O) as a Treatment for Refractory Depression: A Word of Caution. Journal of Clinical Psychopharmacology 40, 517. https://doi.org/10.1097/JCP.0000000000001265 - Yang, C., Hashimoto, K., 2015. Combination of Nitrous Oxide with Isoflurane or Scopolamine for Treatment-resistant Major Depression. Clinical Psychopharmacology and Neuroscience 13, 118–120. <https://doi.org/10.9758/cpn.2015.13.1.118> - Zarate Jr, C.A., 2021. Glutamate modulators and beyond: a neuroscience revolution in the making. *European neuropsychopharmacology: the journal of the European College of Neuropsychopharmacology*, *54*, p.72. - Breivik, H., 2015. Nitrous oxide in oxygen (50: 50) is analgesic that requires optimal inhalation procedure. *Scandinavian Journal of Pain*, *7*(1), pp.35-37. - Kronenberg, G., Schoretsanitis, G., Seifritz, E. and Olbrich, S., 2024. The boon and bane of nitrous oxide. *European Archives of Psychiatry and Clinical Neuroscience*, pp.1-8. - Whizar-Lugo, V.M.M.D., Heredia-Mota, C.M.D. and Camacho, A.M.D., 2017. Back to the Future: Ketamine and Nitrous Oxide in Major Refractory Depression. *J Anesth Crit Care Open Access*, *9*(3), p.00345. - Xiang, Y., Li, L., Ma, X., Li, S., Xue, Y., Yan, P., Chen, M. and Wu, J., 2021. Recreational nitrous oxide abuse: prevalence, neurotoxicity, and treatment. *Neurotoxicity Research*, *39*, pp.975-985. - Gillman, M.A., 2019. Words of caution on using fixed 50% concentrations of nitrous oxide in psychiatry. *Journal of Clinical Psychopharmacology*, *39*(4), pp.421-422. - Terao, T., 2023. Treatments for major depression. *The Lancet*, *401*(10394), pp.2110-2111. - Gillman, M.A., 2024. Study of Antidepressant Actions of Subanesthetic Nitrous Oxide: Importance of Adequate Blinding and Opioid Receptors. *Biological Psychiatry: Global Open Science*, *4*(4). - Gillman, M.A., 2022. What is better for psychiatry: Titrated or fixed concentrations of nitrous oxide?. *Frontiers in Psychiatry*, *13*, p.773190. - Conway, C.R., George, M.S. and Sackeim, H.A., 2017. Toward an evidence-based, operational definition of treatment-resistant depression: when enough is enough. *JAMA psychiatry*, *74*(1), pp.9-10. - Williams, E., Taujanskaite, U., Kamboj, S.K., Murphy, S.E. and Harmer, C.J., 2025. Examining memory reconsolidation as a mechanism of nitrous oxide’s antidepressant action. *Neuropsychopharmacology*, pp.1-9. - Hughes, M.E., 2024. Nitrous Oxide in the Treatment of Depression: A Brief Review. - Williams, Ella, and Philip J. Cowen. "NO LAUGHING MATTER: NITROUS OXIDE AND DEPRESSION." *Biological Psychiatry Global Open Science* (2025): 100551. - Garakani, Amir. "Commentary on Nitrous Oxide: Weighing the Toxic Neuropsychiatric Effects of Abuse Against Potential Therapeutic Benefits for Psychiatric Disorders." *Journal of Psychiatric Practice®* 31, no. 3 (2025): 178-179. |
| 1 | Ineligible because they did not use N2O | - Tadler, S.C., Jones, K.G., Lybbert, C., Huang, J.C., Jawish, R., Solzbacher, D., Kendrick, E.J., Pierson, M.D., Weischedel, K., Rana, N. and Jacobs, R., 2023. Propofol for treatment resistant depression: A randomized controlled trial. medRxiv, pp.2023-09. |
| 4 | Book chapter | - Conway, C.R., Mickey, B.J., Palanca, B.J., Tadler, S.C. and Nagele, P., 2022. Inhaled gases for treatment-resistant major depression. In *Managing Treatment-Resistant Depression* (pp. 481-491). Academic Press. - Hristidis, V.C., Nagele, P., Palanca, B.J., Tadler, S.C., Mickey, B.J., Gott, B.M. and Conway, C.R., 2024. The use of inhaled gases in psychiatry. In *Interventional Psychiatry* (pp. 383-401). Academic Press. - Kamel, L.Y., Quach, D.F., Gott, B.M. and Conway, C.R., 2021. Nitrous Oxide: An Old Compound with Emerging Psychotropic Properties. *New Rapid-acting Antidepressants*, pp.49-61. - Sheldon, R., Schon, F. and Poole, N., 2020. 11 The neuropsychiatry of nitrous oxide. |
| 12 | Other | - A molecular mechanism in the antidepressant effect of ketamine. Neuroscientist. 2024 Apr;30(2):156. doi: 10.1177/10738584241236159. PMID: 38465457. - Antidepressant response to nitrous oxide and cerebral connectivity and brain tissue pulsations. Neuroscientist. 2024 Apr;30(2):156. doi: 10.1177/10738584241236159a. PMID: 38465454. - Demarinis, S., 2021. Persistent loneliness can increase dementia risk. *Explore*, *17*(6), pp.485-486. - Saarreharju, R., 2020. The Shared Behavioral and Biochemical Effects of Rapid-Acting Antidepressants Ketamine and Nitrous Oxide in a Chronic Corticosterone-Induced Animal Model of Depression. - Caplen, S.M., New Treatments for Depression: Ketamine, Propofol, Nitrous Oxide, Psychedelics and Transcranial Magnetic Stimulation. What Is the Evidence?. - Kaar, S.J., Ferris, J., Waldron, J., Devaney, M., Ramsey, J. and Winstock, A.R., 2016. Up: The rise of nitrous oxide abuse. An international survey of contemporary nitrous oxide use. *Journal of psychopharmacology*, *30*(4), pp.395-401. - Zhang, C., Zhang, Y., Li, J., Li, D., Jin, H., Voon, V. and Sun, B., 2020. 10 Deep brain stimulation of the bilateral habenula for treatment resistant depression: preliminary results of six patients. - Shao, X., Yan, D., Kong, W., Sun, S., Liao, M., Ou, W., Zhang, Y., Zheng, F., Li, X., Li, L. and Hu, B., 2023. Brain function changes reveal rapid antidepressant effects of nitrous oxide for treatment-resistant depression: Evidence from task-state EEG. *Psychiatry research*, *322*, p.115072. - Kong, W., Sun, Z., Zhu, J., Li, L., Wang, G., Shao, X., Li, X. and Hu, B., 2025. Alterations in temporal-spatial brain entropy in treatment-resistant depression treated with nitrous oxide: Evidence from resting-state EEG. *Clinical Neurophysiology*. - Li, N., Wang, Z., Huang, J., Sun, S., Zhu, J., Li, X. and Hu, B., 2024, December. Nitrous oxide therapy-induced changes in brain modules of treatment-resistant depression: a randomized controlled study. In *2024 IEEE International Conference on Bioinformatics and Biomedicine (BIBM)* (pp. 3428-3433). IEEE. - Hawkins, Jemma, Lindsey A. Hines, Chris Bonell, Matthew Hickman, Linda Adara, Julia Townson, Rebecca Cannings-John, Laurence Moore, and James White. "Association of volatile substance, nitrous oxide and alkyl nitrate use with mental health in UK adolescents." *The British Journal of Psychiatry* 226, no. 1 (2025): 10-15. - Hadley, Morgan, Alicia Halliday, and James M. Stone. "Association of hallucinogen persisting perception disorder with trait neuroticism and mental health symptoms." *Journal of Psychoactive Drugs* 57, no. 1 (2025): 47-53. |
