# Supplementary material for: Nitrous oxide for the treatment of depression: a systematic review and meta-analysis
Source: eBioMedicine. 2025 Nov 30;122:106023. doi: 10.1016/j.ebiom.2025.106023 (PMC12790589; doi:10.1016/j.ebiom.2025.106023)
Supplement: Supplementary Table S2 [file mmc6.docx]

|  | | | | | | | |  |  |  |
| --- | --- | --- | --- | --- | --- | --- | --- | --- | --- | --- |
| **Study ID** | **Condition** | **Inclusion Age** | **N** | **Comparison Type** | **Study type and Phase** | **N2O Dose** | **Total sessions, duration and frequency** | **Outcome measures** | **Status** |  |
| NCT03736538 | MDD | 18 - 65 | 50 | Placebo controlled (N2O vs. nitrogen/oxygen mixture) | RCT, Phase 1 | 50% | Duration not reported, maximum of four one-hour inhalation sessions as inpatients and 2 booster sessions as outpatients. | Severity and number of AEs, SSI, | Enrolling by invitation |  |
| NCT05357040 | MDD | ≥18 years | 172 | Placebo controlled (N2O vs. oxygen/air) | RCT, Phase 2 | 25%, 50% | 60-minute sessions of 25% or 50% N2O for 4 weeks. | HDRS-21, POMS, CAT-MH, S-STS, VAS | Recruiting |  |
| NCT06430489 | Suicidal ideation | 18 - 50 | 30 | Placebo controlled (N2O vs. medical air) | RCT, Phase 2 | 50% | 60-minutes session, frequency not reported | SSI, C-SSRS, MADRS, PHQ-9, STAI, CGI, | Not yet recruiting |  |
| NCT05710887 | MDD/TRD | 18 - 65 | 50 | Placebo controlled (N2O vs. oxygen/air) | RCT, Phase 2 | 50% | Single 45-minute session | CAT-MH | Not yet recruiting |  |
| NCT06382389 | Depression and major neurocognitive disorder | ≥ 60 | 96 | Placebo controlled (EMONO vs. medical air) | RCT, Phase 2 | 50% | Three sessions: 20-minutes (week 0), 4- minutes (week 1), and 60-minutes (week 2) | CORNELL, GDS scale, CGI, EVIBE, | Not yet recruiting |  |
| NCT06636357 | Suicidal ideation | 18 - 65 | 85 | Placebo controlled (N2O vs. oxygen) | RCT, Phase 2 | 50% | 45-minute session, frequency not reported. | BSS, MADRS, S-VAS, PB-VAS | Recruiting |  |
| NCT05007028 | TRD | 60 - 90 | 60 | Placebo controlled (EMONO vs. medical air) | RCT, - | - | 60-minute session, frequency not reported | MADRS, Brain tissue pulsatility, HDRS-21, QIDS-SR, CGI, VAS, STAI, SSI, YMRS, CADSS, BPRS | Recruiting |  |
| NCT06557642 | Depression | 18 - 40 | 84 | Placebo controlled (ENTONOX vs. medical air) | RCT, - | 50% | 30-minute session, frequency not reported | Memory reconsolidation, Performance on 1) emotional processing tasks, 2) cognitive flexibility tasks, 3) response inhibition task | Recruiting |  |
