# Supplementary material for: Nitrous oxide for the treatment of depression: a systematic review and meta-analysis
Source: eBioMedicine. 2025 Nov 30;122:106023. doi: 10.1016/j.ebiom.2025.106023 (PMC12790589; doi:10.1016/j.ebiom.2025.106023)
Supplement: Supplementary Table S3 [file mmc7.docx]

| **Domain** | **Ketamine** | **N2O** |
| --- | --- | --- |
| Mechanism of action | Non-competitive NMDA receptor antagonist; increases AMPA throughput; engages BDNF-mTOR pathways and synaptogenesis; interaction with opioid pathways. | Non-competitive NMDA receptor antagonist; interacts with opioid receptors, downstream effects on  glutamatergic signalling and neuroplasticity. |
| Formulation and administration | Intravenous racemic ketamine (0.5mg/kg over 30-40 minutes) widely studied; intranasal esketamine (56-84 mg) approved; oral subcutaneous and intramuscular routes under investigation. | Inhaled gas at 25% or 50% concentration, administered for 20-60 minutes via anaesthesia machines, non-rebreathing circuits, or fixed-dose cylinders. |
| Trial phase and state of evidence | Large number of Phase II-III RCTs; pooled n>1000; multiple meta-analyses. . | 7 completed RCTs (n=247); all early phase (Phase I-II), mostly investigating single dosing; largest ongoing RCT (NCT05357040) aims for n=172. |
| Populations studied | Predominantly TRD, often multi-drug resistant; trials also in suicidal depression, BD, adolescents and older adults. | Adults with MDD, TRD and BD; no completed adolescent trials. Pooled results from three trials administering 50% N2O in a single session showed significant reductions in depressive symptoms at 2 hours (pooled MD -2·74, 95% CI: -4·72 to -0·76; p=0·007) and 24 hours (MD -3·32, 95% CI: -5·09 to -1·55; p<0·0001), |
| Effects on depressive symptoms at 2 and/or 24 hours post-treatment | Robust efficacy at 24 hours post-administration: meta-analysis SMD 0·68, 95% CI 0·46-0·90. |  |
| Durability of effect | Declines by 1 week post-administration (SMD 0·49); repeated infusions associated with more sustained responses up to 2-6 weeks post-treatment. | Anti-depressant effects not sustained at 1 week post-inhalation (MD -1·52; 95% CI: -4·07 to 1·03; p=0·24); repeated dosing may extend benefit but evidence remains limited. |
| Adverse events | Dissociation, perceptual disturbances, anxiety, transient increases in blood pressure and heart rate 9dose-releated); low discontinuation rates; abuse potential has led to scheduling. | Generally mild and transient; nausea, vomiting, dizziness, headaches more frequent with 50% N2O; rare AEs with prolonged/recreational exposure include B12 deficiency and neurological deficits. |
| Regulatory status | Acceptable tolerability; discontinuation uncommon; prolonged monitoring required due to dissociation and cardiovascular effects. | Well tolerated in trials; low discontinuation rates; AE profile dose-dependent. |
| Quality of evidence | Larger evidence base but systematic reviews rates “critically low” on AMSTAR-2 due to inadequate bias assessments and selective reporting. | Evidence base remains limited, with small sample sizes, early-phase designs, variable delivery methods and short follow-up. |
| Regulatory status | FDA and EMA approved as a treatment for TRD. | Not approved for psychiatric disorders; widely used for anaesthesia/analgesia; not controlled. |
| Research gaps | Uncertainty about long-term efficacy, safety and comparative effectiveness; biomarkers of response under investigation. | Lack of large, methodologically rigorous RCTs; absence of adolescent trials; uncertainty about repeated and maintenance dosing, and long-term safety. |
