# Supplementary material for: Nitrous oxide for the treatment of depression: a systematic review and meta-analysis
Source: eBioMedicine. 2025 Nov 30;122:106023. doi: 10.1016/j.ebiom.2025.106023 (PMC12790589; doi:10.1016/j.ebiom.2025.106023)
Supplement: Supplementary Material 1 [file mmc8.docx]

("Depression"[MeSH] OR "Depressive Disorder"[MeSH] OR "Major Depressive Disorder" OR "Mood Disorders"[MeSH] OR “depression” OR "depressive disorder" OR "major depressive disorder" OR "mood disorder*" "Treatment-Resistant Depression" OR "Refractory Depression" OR "treatment-resistant depression" OR "refractory depression" OR "difficult-to-treat depression" OR "difficult to treat depression") AND ("Nitrous Oxide"[MeSH] OR "nitrous oxide" OR "laughing gas" OR N2O)
