# Supplementary material for: Nitrous oxide for the treatment of depression: a systematic review and meta-analysis
Source: eBioMedicine. 2025 Nov 30;122:106023. doi: 10.1016/j.ebiom.2025.106023 (PMC12790589; doi:10.1016/j.ebiom.2025.106023)
Supplement: Supplementary file 7 [file mmc1.docx]

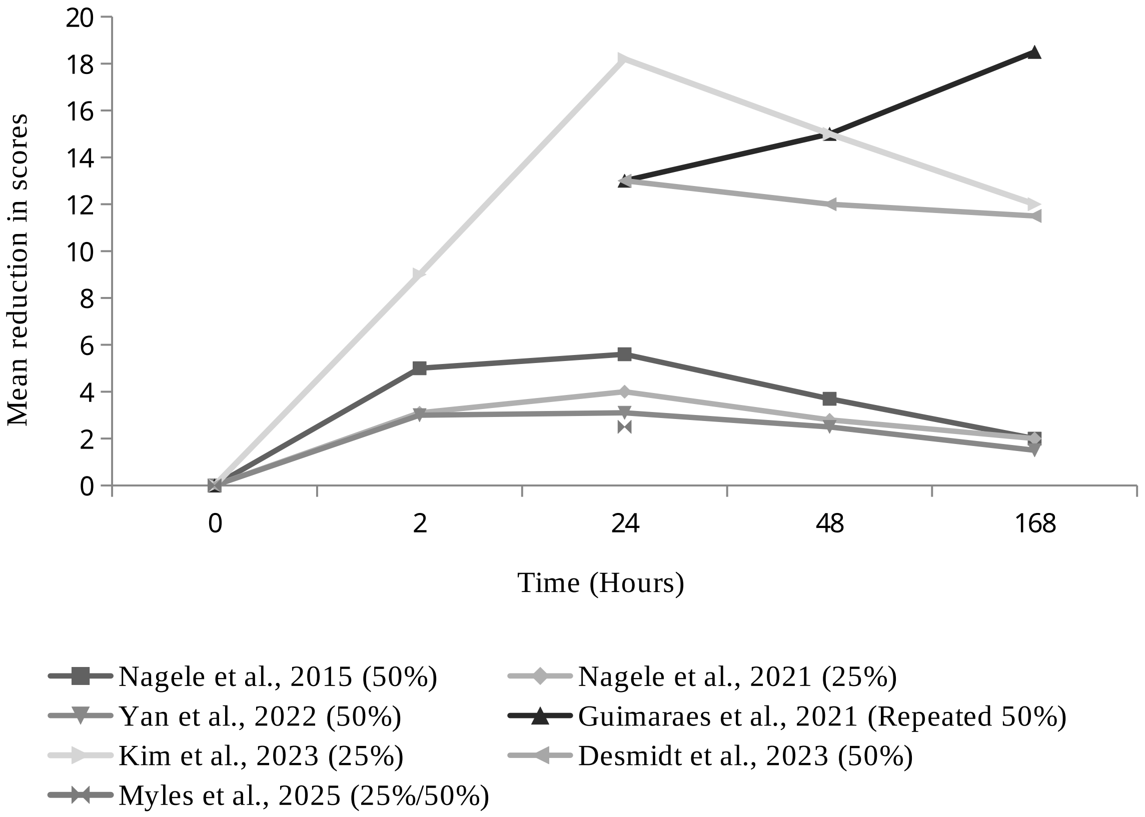

Mean reduction in scores
20
18
16
14
12
10
8
6
4
2
0
0
2
24
48
168
Time (Hours)
Nagele et al., 2015 (50%)
Nagele et al., 2021 (25%)
Yan et al., 2022 (50%)
Guimaraes et al., 2021 (Repeated 50%)
Kim et al., 2023 (25%)
Desmidt et al., 2023 (50%)
Myles et al., 2025 (25%/50%)
